# Whole-Exome Sequencing of Bronchial Epithelial Cells Reveals a Genetic Print of Airway Remodelling in COPD

**DOI:** 10.3390/biomedicines10071714

**Published:** 2022-07-15

**Authors:** Adeline Germain, Jeanne-Marie Perotin, Gonzague Delepine, Myriam Polette, Gaëtan Deslée, Valérian Dormoy

**Affiliations:** 1 Inserm, P3Cell UMR-S1250, Université de Reims Champagne-Ardenne, SFR CAP-SANTE, 51092 Reims, France; adeline.germain@univ-reims.fr (A.G.); jmperotin-collard@chu-reims.fr (J.-M.P.); gdelepine@chu-reims.fr (G.D.); myriam.polette@univ-reims.fr (M.P.); gdeslee@chu-reims.fr (G.D.); 2 Service de Pneumologie, CHU Reims, Hôpital Maison Blanche, 51092 Reims, France; 3 Service de Chirurgie Thoracique, CHU Reims, Hôpital Maison Blanche, 51092 Reims, France; 4 Laboratoire de Biopathologie, CHU Reims, Hôpital Maison Blanche, 51092 Reims, France

**Keywords:** whole-exome sequencing, airway epithelial cells, COPD

## Abstract

The remodelling of the airways is a hallmark of chronic obstructive pulmonary disease, but it is highly heterogeneous and erratically distributed in the airways. To assess the genetic print of remodelling in chronic obstructive pulmonary disease (COPD), we performed a comparative whole-exome sequencing analysis on microdissected bronchial epithelia. Lung resections from four non-COPD and three COPD subjects (ex-smokers and current smokers) were formalin-fixed paraffin-embedded (FFPE). Non-remodelled and remodelled bronchial epithelia were isolated by laser microdissection. Genomic DNA was captured and sequenced. The comparative quantitative analysis identified a list of 109 genes as having variants in remodelled epithelia and 160 genes as having copy number alterations in remodelled epithelia, mainly in COPD patients. The functional analysis highlighted cilia-associated processes. Therefore, bronchial-remodelled epithelia appeared genetically more altered than non-remodelled epithelia. Characterizing the unique molecular print of airway remodelling in respiratory diseases may help uncover additional factors contributing to epithelial dysfunctions, ultimately providing additional targetable proteins to correct epithelial remodelling and improve lung function.

## 1. Introduction

When applied to cases of chronic obstructive pulmonary disease (COPD), whole-exome sequencing (WES) analysis identified a few candidate genes in lung function alterations, emphysema or functional variants in resistant smokers [1,2,3,4,5].

The molecular alteration of airway basal cells, which are the progenitors of secretory and ciliated cells, is central to the pathogenesis of COPD [6,7,8]. We previously demonstrated the dysregulation of primary cilia associated with the presence of epithelial remodelling in lung tissues or airway epithelial cells obtained from COPD patients [9,10].

Considering that the observed remodelling may be transient in non-COPD patients, while it may persist in COPD patients, we thought to investigate a genetic print of epithelial remodelling in COPD in this study. Therefore, we isolated non-remodelled and remodelled epithelia by laser microdissection, and we performed differential whole-exome sequencing on both non-COPD and COPD patients.

## 2. Materials and Methods

### 2.1. Human Subjects

**Figure 1:**
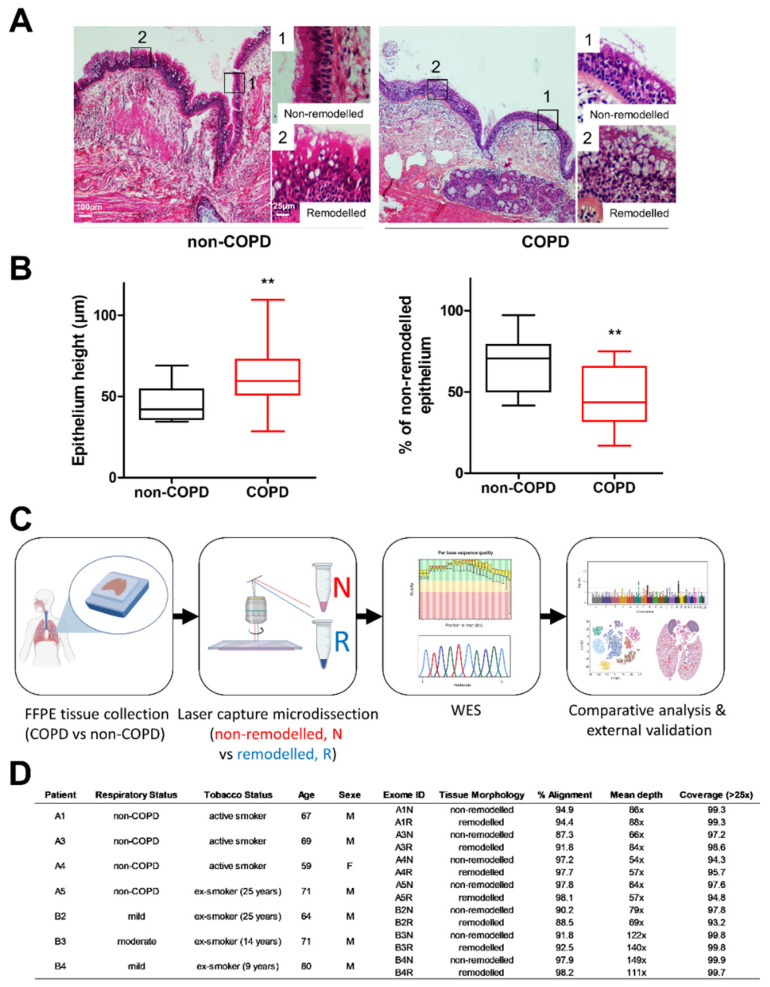
Epithelial remodelling and study design for laser microdissection. (**A**) Examples of HES-stained bronchial epithelia of non-COPD and COPD patients showing non-remodelled and remodelled epithelia. Boxed areas are shown as magnifications (1, non-remodelled epithelia; 2, remodeled epithelia). (**B**) Box plots (mean and IQR) showing bronchial epithelia height and percentage of non-remodelled epithelia in 17 non-COPD smokers or ex-smokers and 17 mild-moderate COPD smokers or ex-smokers. **, *p* < 0.01 COPD vs. non-COPD. (**C**) Schematic workflow for the collection and genetic analysis of non-remodelled (N) and remodelled (R) non-COPD (**A**) and COPD (**B**) epithelia. (**D**) Table summarizing the main clinical features of the patients (A1, A3, A4, A5: non-COPD; B2, B3, B4: COPD) and NGS quality control after exome sequencing.

Patients scheduled for lung resection for the treatment of cancer (University Hospital of Reims, France) were prospectively recruited (*n* = 7) following standards established and approved by the institutional review board of the University Hospital of Reims, France (IRB Reims-CHU 20110612). Informed consent was obtained from all patients. Patients with asthma, cystic fibrosis, bronchiectasis or pulmonary fibrosis were excluded. At inclusion, age, sex, smoking history and pulmonary function test results were recorded (Figure 1). The lung function was significantly lower in the group of COPD patients (FEV1%: 60.3 ± 6 vs. 102 ± 11.4, *p* < 0.05). Ex-smokers were considered if they were in a state of withdrawal longer than 6 months. COPD was defined by post-bronchodilator FEV1/FVC< 0.7 [11]. The severity of COPD was determined by spirometric classification. The epithelium height and remodelling were evaluated on 17 non-COPD and 17 mild and moderate COPD patients described in detail in [9].

### 2.2. Immunostaining

Immunohistochemistry was performed on formalin-fixed paraffin-embedded (FFPE) lung tissues distant from the tumours as previously described [9]. Five μm sections were processed for H&E staining and observed on a microscope (×20) to confirm the presence of bronchi. Micrographs were acquired by AxioImageur Zeiss (20 × Ph) with ZEN software (8.1, 2012) and processed with ImageJ (National Institutes of Health) for analysis. Epithelium height was measured on each micrograph via ImageJ by averaging between the thickest and the thinnest epithelium for each field. The remodelling features included basal cell hyperplasia, secretory cell hyperplasia and squamous metaplasia, which are assessed by a histologist (VD).

### 2.3. Pre-Processing of FFPE Blocs

FFPE lung sections were cut on a Leica microtome (Leica Biosystems Inc., Buffalo Grove, IL, USA) in sterile and DNA-free conditions. They were placed on a MembraneSlide 1.0 PEN (Carl Zeiss Microscopy GmbH, Göttingen, Germany), deparaffinized and then stained for the nuclei to facilitate tissue morphology visualization according to the following steps: successive baths in xylene (2.5 min), EtOH 100% (1 min), EtOH 70% (1 min), milliQ water (1 min), Harris hematoxylin ½ (20 s), milliQ water (1 min), EtOH 70% (1 min) and EtOH 100% (1 min) before air drying (5 min) and storing at room temperature in a sterile container until microdissection.

### 2.4. Laser Capture Microdissection (LCM)

The isolation of non-remodelled and remodelled epithelia from bronchi (large airways, 3rd to 5th generations) was performed on a PALM^®^ MicroBeam system (Zeiss, Bernried, Germany). A normal epithelium was defined as a pseudostratified epithelium: (a) presenting the three main cell types (basal, ciliated and goblet cells); (b) lacking hyperplasia or metaplasia; and (c) showing at least 50% of ciliated cells at the surface. The tissues were separately cut from the parenchyma with a software-controlled laser beam (PALM RoboSoftware 4.6) at a 20× cutting objective. The sections containing only epithelia were catapulted by a laser pulse, without risks of contamination (non-contact laser pressure catapulting process), into the cap of a 0.2 µL microtube containing 20 µL of the incubation buffer (Maxwell^®^ 16 FFPE Plus LEV DNA Purification Kit, Promega Corporation, Madison, WI, USA). Every 45 min, the microtube was centrifugated to collect the sections at the bottom of the microtube, and the LCM procedure was repeated several times. At the end of the procedure, the microtubes’ contents were transferred to a 1.5 mL microtube with 0.1 volumes of proteinase K (20 mg/mL) and incubated in a dry bath for 24 h at 70 °C.

### 2.5. DNA Extraction from FFPE Sections

After proteinase K treatment, two volumes of the lysis buffer were added. DNA extraction was performed on a Maxwell^®^ 16 instrument according to the manufacturer kit procedure (Maxwell^®^ 16 FFPE Plus LEV DNA Purification Kit, Promega Corporation, Madison, WI, USA). DNA was eluted in 50 µL of nuclease-free water and quantified on a Qubit^®^ fluorometer according to the Qubit^®^ dsDNA HS Assay Kit procedure (LifeTechnologies, Carlsbad, CA, USA) on 1 µL of DNA. Two µL were used for DNA quality control by qPCR. The DNA samples were stored at −20 °C before sequencing. qPCR for DNA quality, targeted exome sequencing and bioinformatics are described in Supplementary Materials: Detailed Material and Methods.

### 2.6. RNAseq Data Analysis

A previously published dataset of gene expression of whole-lung tissue samples obtained from 108 non-COPD and 133 COPD patients was collected from datasets available online (GEO database; http://www.ncbi.nlm.nih.gov/geo; accession numbers: GSE47460). Severe and very severe COPD patients were excluded. The expressions of the genes of interest identified in variants (SNV and Indel) and copy number alteration (CNA) analyses were extracted for each patient sample and expressed as log2(fold-change) in the COPD group compared to non-COPD. The GSE plot contains all the genes of the gene set. The *y*-axis is the log10(FDR).

### 2.7. Single-Cell Sequencing Analysis

The published dataset can be found on www.lungcellatlas.org. We retained cell clustering based on the original studies and considered only subjects free of respiratory disease for the extraction of the single-cell transcript prints of the genes of interest in the lung epithelial and non-epithelial cells [12].

### 2.8. Statistics

Statistical analyses were performed using SPSS v24. The clinical and experimental data were analysed by Student’s t-tests. The expressions of the genes of interest were analysed using paired t-tests, which were applied to the log2-transformed transcriptomic data; the Benjamini and Hochberg false discovery rate (FDR) correction was applied; *p* < 0.05 was considered significant.

## 3. Results

The epithelial remodelling in non-COPD and COPD bronchi was assessed by standard histological analysis (Figure 1A,B). The bronchial epithelium height was significantly higher in COPD patients (46.3 ± 2.6µm in non-COPD vs. 63.7 ± 4.9µm in COPD; *n* = 17/17, *p* < 0.01), and the percentage of the non-remodelled epithelium was lower (67.6 ± 3.8% of non-remodelled epithelia in non-COPD vs. 47.2 ± 4.4% in COPD; *n* = 17/17, *p* < 0.01). Nonetheless, non-remodelled and remodelled epithelial areas coexisted and intertwined in the bronchial tree (Figure 1A,B). To investigate the potential local genetic alterations of epithelial cells, we performed a laser capture microdissection of bronchial epithelia on four non-COPD and three COPD FFPE lung tissues to separately extract the DNA from the non-remodelled and remodelled areas, and to comparatively analyse the whole exomes (Figure 1C,D and Supplementary Materials: Figures S1–S4).

**Figure 2:**
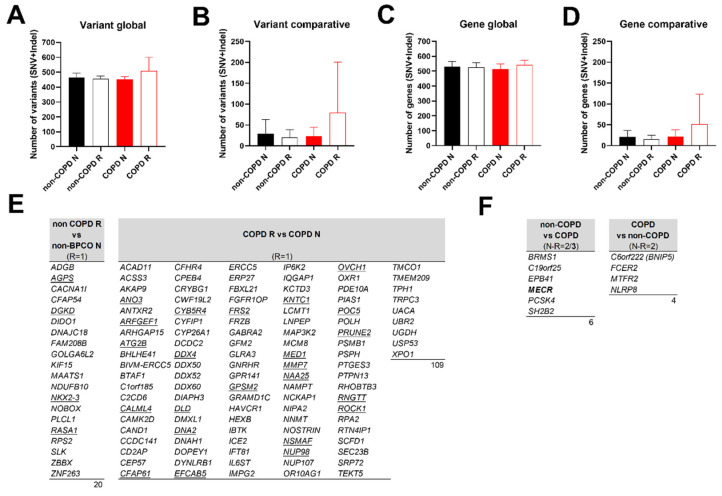
Identification of genetic variations in COPD microdissected remodelled epithelia. Histograms (mean +/− SEM) showing the number of identified positions or genes with variants according to respiratory status and tissue morphology (N: non-remodelled; R: remodelled). (**A**) Identical positions between N and R were retained (**B**) or removed. (**C**) Identical genes between N and R were retained (**D**) or removed. (**E**) Potential frequent genes variants of remodelled non-COPD and COPD tissue. Genes presenting an SNV or an Indel in the remodelled tissue and absent in the non-remodelled tissue for the same position. Non-COPD R vs. non-COPD N: variants in non-COPD R not found in non-COPD N. COPD R vs. COPD N: variants in COPD R not found in COPD N. These variants were only found in one R sample (R = 1). Underlined genes are for the variants assumed to have high (disruptive) impact in the protein. (**F**) Lists of potential frequent gene variants of COPD susceptibility. Genes with identical SNV/Indel between non-remodelled and remodelled tissue for 2 or 3 samples (N-R = 2/3 (bold)). Non-COPD vs. COPD: variants in non-COPD not found in COPD. COPD vs. non-COPD: variants in COPD not found in non-COPD. See Supplementary Materials: Table S1 for impact description.

Considering the number of identified variants (single nucleotide variant (SNV), insertion or deletion), there was no difference between non-COPD and COPD epithelia in association with remodelling (global analysis: 465.5 ± 13.6 in non-COPD non-remodelled epithelia (N) vs. 456 ± 9.1 in non-COPD remodelled epithelia (R) vs. 453 ± 10.5 in COPD N vs. 507 ± 52.8 variants in COPD R) (Figure 2A). Setting the reference as unique variants between non-COPD and COPD patients (comparative analysis) did not change the outcome (29.8 ± 16.9 in non-COPD N vs. 20.5 ± 9.3 in non-COPD R vs. 23.7 ± 12.2 in COPD N vs. 79.3 ± 69.8 variants in COPD R) (Figure 2B). Looking at the impacted genes provided similar results: 530.8 ± 16.5 in non-COPD N vs. 524.5 ± 15.6 in non-COPD R vs. 515 ± 18.8 in COPD N vs. 541.7 ± 18.5 altered genes in COPD R in the global analysis (Figure 2C); 20.5 ± 7.8 in non-COPD N vs. 15.3 ± 5 in non-COPD R vs. 21.7 ± 9.7 in COPD N vs. 51.3 ± 41.3 altered genes in COPD R in the comparative analysis (Figure 2D).

Although the characterization of the variants did not significantly distinguish remodelled epithelia in COPD patients, we identified a list of 129 genes of interest with variants in remodelled epithelia only (Figure 2E and Supplementary Materials: Table S1), and 10 genes of interest having identical variants in remodelled and non-remodelled epithelia found in more than two samples (Figure 2F and Supplementary Materials: Table S1). The classification of the severity of the variant consequence revealed similar functional effects between non-COPD and COPD comparative analyses (4/20 high impact vs. 23/109). Fifteen genes harboured somatic mutations in more than 5% of affected lung cancer (adenomas/adenocarcinomas and squamous cell neoplasms) in The Cancer Genome Atlas (TCGA). Interestingly, gene ontology analysis revealed functional enrichments in cilia movement (strength = 0.97, FDR = 0.0455), microtubule-based processes (strength = 0.55, FDR = 0.0472) and cell projection assembly (strength = 0.55, FDR = 0.048) (Supplementary Materials: Figure S5).

**Figure 3:**
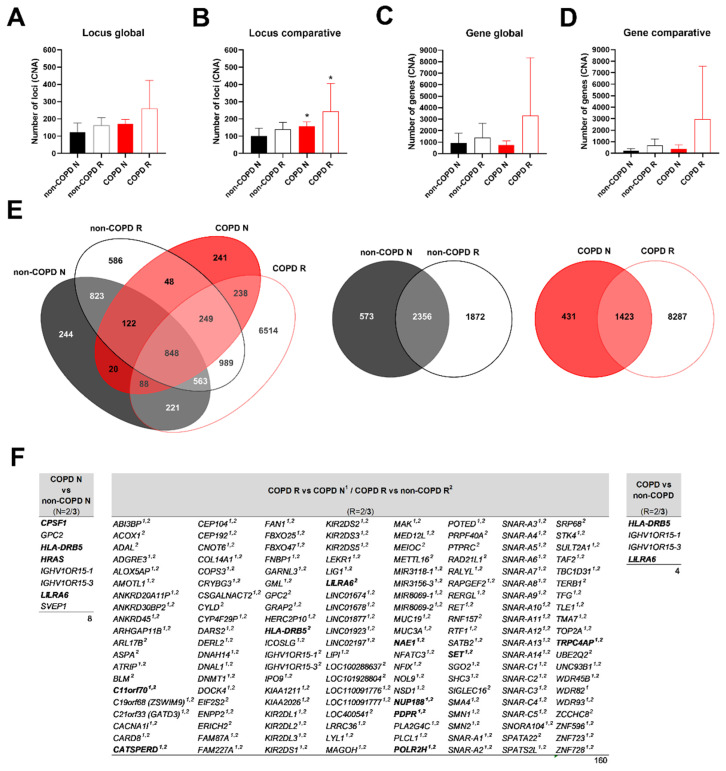
Identification of copy number alterations in COPD microdissected remodelled epithelia. Histograms (mean ± SEM) showing the number of identified loci or genes with CNA according to respiratory status and tissue morphology (N: non-remodelled; R: remodelled). (**A**) Identical loci (same start and end positions) between N and R were retained (**B**) or removed. (**C**) Identical genes between N and R were retained (**D**) or removed. *, *p* < 0.05 COPD N vs. non-COPD N, and COPD R vs. non-COPD N. (**E**) Venn diagrams of overlapping genes with CNA in the global repartition. (**F**) Lists of potential frequent genes with CNA in COPD epithelia (1; COPD R vs. COPD N; 2, COPD R vs. non-COPD R). Genes presenting a CNA for 2 or 3 COPD samples (N or R = 2/3 (bold)). See Supplementary Materials: Figure S6 for copy number description.

Considering the number of loci where copy number alterations (CNA) were found, there was no difference between non-COPD and COPD epithelia in association with the remodelling in the global analysis (123 ± 26 in non-COPD N vs. 162.3 ± 22.1 in non-COPD R vs. 171.7 ± 14.5 in COPD N vs. 258.7 ± 95 loci in COPD R) (Figure 3A). Setting the reference as unique CNA between non-COPD and COPD patients (comparative analysis) revealed a significant 1.6-fold increase in loci with CNA in COPD N and a significant 2.4-fold increase in loci with CNA in COPD R compared to non-COPD N (100.3 ± 23.3 in non-COPD N vs. 139.5 ± 19.9 in non-COPD R vs. 157.7 ± 14.3 in COPD N vs. 244.7 ± 93.2 loci in COPD R; *p* < 0.05) (Figure 3B). Looking at the impacted genes provided similar results: 923.6 ± 422 in non-COPD N vs. 1402 ± 618.5 in non-COPD R vs. 735 ± 215.6 in COPD N vs. 3322 ± 2905 altered genes in COPD R in the global analysis (Figure 3C), and 221.5 ± 94.8 in non-COPD N vs. 694.8 ± 274.2 in non-COPD R vs. 380 ± 202.5 in COPD N vs. 2967 ± 2643 altered genes in COPD R in the comparative analysis (Figure 3D). Only 848 genes with CNA were found in common, but 81.7% of CNA were unique in COPD R compared to COPD N, while 39% were unique in non-COPD R compared to non-COPD N (Figure 3E).

We identified 163 genes of interest as having CNA in at least two samples where most of them are only found in COPD R (Figure 3F and Supplementary Materials: Figure S6). Ten genes presented can in the three COPD remodelled samples (Figure 3F, genes in bold): C11orf70, CATSPERD, HLA-DRB5, LILRA6, NAE1, NUP188, PDPR, POLR2H, SET and TRPC4AP. The functional analysis highlighted the compartment nuclear lumen (strength = 0.39, FDR = 0.0418) (Supplementary Materials: Figure S7). Therefore, remodelled epithelia in non-COPD tissues genetically resembled non-remodelled epithelia, whereas remodelled epithelia in COPD tissues genetically differed from remodelled non-COPD and non-remodelled COPD epithelia.

Interestingly, variants acanCNA were jointly identified in 98 genes (Supplementary Materials: Figure S8). Furthermore, the global analysis of the genes displaying variants acanor CNA in COPD R (n = 269) identified an enrichment in the Reactome cell cycle (strength = 0.45, FDR = 0.0416), or UniProt keywords acetylation (strength = 0.22, FDR = 0.0049) and alternative splicing (strength = 0.12, FDR = 0.00038) (Supplementary Materials: Figure S9). Considering the 202 (among the 269) unique proteins found in all the networks, a comparative analysis with cilia-associated proteins from UniProt revealed that 23 proteins are present (14 with variants ancan with CNA) representing more than 10% of the proteins in the networks (Supplementary Materials: Figures S5, S7 and S9). Among these genes, 182 were detected in a dataset comprising COPD whole-lung tissues (108 non-COPD and 133 COPD patients): 33 were significantly differentially deregulated, including 15 variants (Supplementary Materials: Figure S10A). We analysed the localization of the proteins and the expression of the transcripts in airway epithelial and non-epithelial cells of the five most deregulated genes all found in the genes of interest having CNA (RET, COL14A1, NSD1, ZNF728 and AMOTL1; Supplementary Materials: Figure S10). Interestingly, COL14A1 and AMOTL1 are preferentially localized and expressed in basal airway epithelial cells.

## 4. Discussion

Since the advent of NGS, numerous global approaches have been tested to characterize genetic variants or deregulated genes in the context of respiratory diseases such as COPD. Nonetheless, the heterogeneity of the clinical, cellular and molecular features restricted the identification of universal biomarkers [13]. We partially circumvented this difficulty with an experimental strategy focused on the analysis of the potential genetic alterations associated with the epithelial remodelling in COPD (mild/moderate) smokers (ex/active) compared with non-COPD smokers (ex/active). Therefore, we performed the first whole-exome sequencing in remodelled and non-remodelled bronchial epithelia. Although we identified a few variants that may be involved in remodelling processes, we unveiled a genetic print made of unique CNA in remodelled COPD epithelia. We provide here the first experimental clue comforting the concept that morphohistologic remodelling is only transient in non-COPD airways, while a genetic alteration may crystallize the remodelling in COPD airways. Our approach also highlighted the potential genes associated with COPD without considering epithelial remodelling.

The functional analysis of our guided approach pointed to a genetic alteration of the cilia that may extend further than what were already identified as cilia regulators. Since COPD exome and transcriptome sequencing often incriminated cilia-associated genes [1,3,14,15], the identification, the molecular characterization and the clinical validation of these key actors appeared to be highly significant in respiratory research. Additionally, we unveiled the genetic alterations of two hits that were previously suggested as a useful biomarker of smokers at risk of developing COPD (COL14A1, [16]) and emphysema-associated regulator (AMOTL1, [17]). Delving deeper into the characterization of variants and CNA in lung cancer may provide additional molecular insights into the relationship between COPD and lung cancer. Notably, a variant of PRUNE2 was identified in the remodelling areas of COPD patients, and approximately 10% of lung cancer patients were affected by a mutation of PRUNE2. This tumour suppressor gene was well characterized in human prostate cancer, but its potential involvement in the lung has not been investigated [18].

Our study was limited by the low number of patients, but the technical steps, including laser microdissection of the biological material, required a tremendous amount of time, and the quality of the FFPE lung tissues that were necessary for NGS greatly reduced the number of samples that may be included in the analysis. Although we focused here on the exomes, it would be interesting to test whether the transcriptomic signature may identify COPD molecular epithelial remodelling features. Finally, we collected exclusively bronchial epithelia; thus, a complementary analysis should be carried out on bronchiolar and alveolar epithelia. Exploring lung areas surrounded by inflammatory cells and other resident cells may tackle the comparative genetic alterations of additional COPD features, such as small airway remodelling or emphysema.

Airway epithelial remodelling is a major component of respiratory diseases that is highly heterogeneous and similarly found in homeostatic lungs. Dissociating non-remodelled and remodelled areas in the cellular and molecular analysis may provide a pivotal pitch in the understanding of this hallmark to impede its progress.

## Data Availability

All data generated or analysed during the current study are available from the corresponding author on reasonable request. In addition, the dataset comprising the 14 BioSamples is deposited in SRA with the BioProject ID: PRJNA826715.

## Supplementary Materials

The following supporting information can be downloaded at: https://www.mdpi.com/article/10.3390/biomedicines10071714/s1, Detailed material and methods; Table S1: List of SNVs and Indels; Figure S1: Sequencing data of non-COPD non-remodelled samples: major results reported for A1N, A3N, A4N and A5N; Figure S2: Sequencing data of non-COPD remodelled samples: major results reported for A1R, A3R, A4R and A5R.; Figure S3: Sequencing data of COPD non-remodelled samples: major results reported for B2N, B3N and B4N.; Figure S4: Sequencing data of COPD remodelled samples: major results reported for B2R, B3R and B4R; Figure S5: Protein network analysis related to the genes of interest having variants in remodelled COPD epithelia; Figure S6: Heat map of CNA identified in microdissected epithelia; Figure S7: Protein network analysis related to the genes of interest having CNA in at least two samples in remodelled COPD epithelia; Figure S8: Heat map of variants having CNA identified in microdissected COPD epithelia; Figure S9: Protein network analysis related to the list of unique genes displaying variants and/or CNA in remodelled COPD epithelia; Figure S10: Exploration of gene hits associated with remodelling in lung and COPD public data.

## Abbreviation

AMOTL1	Angiomotin-like protein 1
CNA	Copy number alteration
COL14A1	Collagen Type XIV alpha 1 chain
COPD	Chronic obstructive pulmonary disease
FDR	False discovery rate
FFPE	Formalin-fixed paraffin-embedded
GEO	Gene expression omnibus
LCM	Laser capture microdissection
NGS	Next-generation sequencing
TCGA	The Cancer Genome Atlas
SNV	Single nucleotide variant
WES	Whole-exome sequencing
