# Supplementary material for: Whole-Exome Sequencing of Bronchial Epithelial Cells Reveals a Genetic Print of Airway Remodelling in COPD
Source: Biomedicines. 2022 Jul 15;10(7):1714. doi: 10.3390/biomedicines10071714 (PMC9313052; doi:10.3390/biomedicines10071714)
Supplement: Supplementary file 1 [file biomedicines-10-01714-s001.zip › biomedicines-1797878-supplementary.pdf]

Supplementary Materials

Whole exome sequencing of bronchial epithelial cells reveals a genetic print of airway remodelling in COPD

Adeline Germain, Jeanne-Marie Perotin, Gonzague Delepine, Myriam Polette, Gaëtan Deslée, Valérian Dormoy

Table of content

1. **Detailed material and methods** 2
   1.1. **qPCR for DNA quality** 2
   1.2. **Targeted exome sequencing** 2
   1.3. **Bioinformatics** 2
2. **Supporting information Tables** 4
   Table S1. List of SNVs and Indels 4
3. **Supporting information Figures** 13
   Figure S1. Sequencing data of non-COPD non-remodelled samples: major results reported for A1N, A3N, A4N, and A5N. 13
   Figure S2. Sequencing data of non-COPD remodelled samples: major results reported for A1R, A3R, A4R, and A5R. 14
   Figure S3. Sequencing data of COPD non-remodelled samples: major results reported for B2N, B3N, and B4N. 15
   Figure S4. Sequencing data of COPD remodelled samples: major results reported for B2R, B3R, and B4R. 16
   Figure S5. Protein network analysis related to the genes of interest having variants in remodelled COPD epithelia. 17
   Figure S6. Heat map of CNA identified in microdissected epithelia. 18
   Figure S7. Protein network analysis related to the genes of interest having CNA in at least two samples in remodelled COPD epithelia. 19
   Figure S8. Heat map of variants/CNA identified in microdissected epithelia. 20
   Figure S9. Protein network analysis related to the list of genes displaying variants and/or CNA in remodelled COPD epithelia. 21
   Figure S10. Exploration of gene hits associated with remodelling in lung and COPD public data. 22
4. **Additional References** 23

1. Detailed material and methods

1.1. qPCR for DNA quality

DNA quality was assessed on 0.5 ng with the Agilent NGS FFPE QC Kit (Agilent technologies, Santa Clara, USA) according to the manufacturer kit procedure on a real-time PCR machine. A ΔCq integrity score for the sample was determined by comparing SYBR Green qPCR results using primer set A producing a 42-bp amplicon with the results using primer set B, which targets the same DNA region but produces a 123-bp amplicon. The integrity score was normalized using the provided human reference DNA sample to generate the normalized DNA integrity score (ΔΔCq). Only DNA samples with a ΔΔCq<3 (optimal quality control pass qualification) were sequenced.

1.2. Targeted exome sequencing

Library preparation, exome capture, sequencing and data analysis have been done by IntegraGen SA (Evry, France). Genomic DNA was captured using Twist Human Core Exome Enrichment System (Twist Bioscience) + IntegraGen Custom. For detailed explanations of the process, see Gnirke's publication in Nature Methods [1]. Sequence capture, enrichment and elution were performed according to the manufacturer's instruction and protocols (Twist Bioscience) without modification except for library preparation performed with NEBNext® Ultra II DNA Library Prep kit for Illumina® (New England Biolabs®). For library preparation, 150 ng of each genomic DNA were fragmented by sonication and purified to yield fragments of 150-200 bp. Paired-end adaptor oligonucleotides from the NEB kit were ligated on repaired, a-tailed fragments then purified and enriched by 7 PCR cycles. Five hundred ng of these purified Libraries were then hybridized to the Twist oligo probe capture library for 16 hr in a singleplex reaction. After hybridization, washing, and elution, the eluted fraction was PCR-amplified with 8 cycles, purified and quantified by qPCR to obtain a sufficient DNA template for downstream applications.

Each eluted-enriched DNA sample was then sequenced on an Illumina HiSeq 4000 or NovaSeq 6000 as Paired-End (75-100 reads). Image analysis and base calling were performed using Illumina Real-Time Analysis with default parameters.

1.3. Bioinformatics

Sequence reads were mapped to the human genome build GRCh38.p12 (RefSeq assembly accession: GFC_000001405.38) by using the Burrows-Wheeler Aligner (BWA) tool. The duplicate reads (e.g., paired-end reads in which the insert DNA molecules had identical start and end locations in the Human genome) were removed (sambamba tools). Variant calling for the identification of SNVs (Single Nucleotide Variations) and small insertions/deletions (up to 110 bp), was performed via the

Broad Institute's GATK Haplotype Caller GVCF tool (GATK 3.7) for constitutional DNA and via the Broad Institute's MuTect tool (2.0, --max_alt_alleles_in_normal_count=2; --max_alt_allele_in_normal_fraction=0.04) for somatic DNA. An in-house, post-processing was applied to filter out candidate somatic mutations that were more consistent with artefacts or germline mutations. Only somatic mutations considered as PASS or t_lod_fstar with a somatic score of at least 1 were retained. The somatic score was calculated for each mutation ranging from 1 to 30 with a score of 30 standing for the highest confidence. This score takes into account the frequency and counts of mutated alleles in both samples to minimize false-positive variations. Mutations with a QSS score below 20 and a VAF of tumour < 0.02 were removed. Ensembl's VEP (Variant Effect Predictor, release VEP 95.1) program processed variants for further annotation. This tool annotated variants, determined the effect on relevant transcripts and proteins, and predicted the functional consequences of variants. Moreover, an in-house database enabled filtering out sequencing artefacts.

Bioconductor DNACopy package was used to investigate genomic copy number aberrations (CNA) (e.g., copy number gains and copy number losses), by comparing the normal DNA exome data to a reference sample pool. It implemented the circular binary segmentation (CBS) algorithm to segment DNA copy number data. All changes were annotated with the catalogue of the Database of Genomic Variants (DGV) to provide a comprehensive summary of structural variation in the human genome.

Variants were not considered if they were synonymous variants, read depth<10, GQ<20 (genotype quality score), VAF<20%, frequency>0.5 in one external databases (1000 Genomes, gnomAD or Kaviar) or not sequenced in related sample (N vs R). CNA were not considered when the copy number was between 1.51 and 2.49. Due to multiples hits of A4 reads (female) on Y chromosome, X and Y variants and CNA were removed from the analysis. A paired analysis setting the non-remodelled epithelium as a reference was performed for each patient. This was defined as the "comparative" analysis, whereas the "global" analysis corresponded to the non-paired analysis.

2. Supporting information Tables

Table S1. List of SNVs and Indels

Gene	Chr	Position[1]	Base ref	Base alt	Status	Variant class	Consequence[2]	Impact[3]	%VAF	Depth	rs ID	Mutated in lung cancer[4]
ACAD11	3	132626829	G	A	B2R: HTZ	SNV	intron variant	unknown	36	11	rs1193519189	
ACSS3	12	81139279	T	TTTAGTTGCA	B2R: HTZ	INS	intron variant	unknown	20	15		
		81139282	A	C	B2R: HTZ	SNV	intron variant	unknown	21	14	rs1477571932	
ADGB	6	146733104	A	ATGT	A1R: HTZ	INS	intron variant	unknown	42	19		
AGPS	2	177497760	AC	A	A3R: HTZ	DEL	frameshift variant	high	23	17		
		177497763	A	T	A3R: HTZ	SNV	stop gained and splice region variant	high	25	16		
		177497768	A	T	A3R: HTZ	SNV	splice region variant and intron variant	low	33	18	rs1183233931	
AKAP9	7	91980341	T	TA	B2R: HTZ	INS	intron variant	unknown	21	23		7.3%
		92095045	G	A	B2R: HTZ	SNV	missense variant	moderate	23	13		
ANO3	11	26598415	A	AAAATATAAAAATGATAAT T	B2R: HTZ	INS	stop gained and frameshift variant	high	23	17		5.8%
ANTXR2	4	79977726	TTCCCA	T	B2R: HTZ	DEL	intron variant	unknown	21	23		
ARFGEF1	8	67219555	A	C	B2R: HTZ	SNV	stop gained	high	20	10		
ARHGAP1 5	2	143767981	C	T	B2R: HTZ	SNV	splice region variant and intron variant	low	20	15	rs1373810363	
		143767983	C	T	B2R: HTZ	SNV	splice region variant and intron variant	low	20	15	rs1475234853	
ATG2B	14	96329618	TGCCAGTACCTATAAA	T	B2R: HTZ	DEL	inframe deletion and splice region variant	moderate	20	15		
		96329635	CTATTATAAAAAATGCACC A	C	B2R: HTZ	DEL	splice acceptor variant and intron variant	high	20	15		
BHLHE41	12	26122566	T	TCAGCAGCGCGGCCGCGG CGGCAGGGTCGGGCCC	B4R: HTZ	INS	inframe insertion	moderate	58	12	rs1367916606	
BIVM-ERCC5	13	102868282	T	TTTATCAA	B2R: HTZ	INS	intron variant	unknown	30	13	rs772409511	
BRMS1	11	66333261	G	A	A3N : HTZ	SNV	downstream gene variant	unknown	44	38	rs138760461	
		66333261	G	A	A3R: HTZ	SNV	downstream gene variant	unknown	49	57	rs138760461	
		66333261	G	A	A5N : HTZ	SNV	downstream gene variant	unknown	41	53	rs138760461	
		66333261	G	A	A5R: HTZ	SNV	downstream gene variant	unknown	46	32	rs138760461	
BTAF1	10	91959880	A	ATCATCT	B2R: HTZ	INS	inframe insertion and splice region variant	moderate	52	21		

C1orf185	1	51112579	A	AT	B2R: HTZ	INS	intron variant	unknown	27	22	rs1295906336	
		51112584	A	C	B2R: HTZ	SNV	intron variant	unknown	28	21	rs1311411755	
		51112589	TTC	T	B2R: HTZ	DEL	intron variant	unknown	25	20	rs1286431924	
C2CD6	2	201572042	T	TAAGTCTCGTTAATA	B2R: HTZ	INS	intron variant	unknown	25	16		
C6orf222 (BNIP5)	6	36330539	G	A	B3N: HTZ	SNV	missense variant	moderate	46	126	rs74561471	
		36330539	G	A	B3R: HTZ	SNV	missense variant	moderate	52	198	rs74561471	
		36330539	G	A	B4N: HTZ	SNV	missense variant	moderate	53	171	rs74561471	
		36330539	G	A	B4R: HTZ	SNV	missense variant	moderate	47	103	rs74561471	
C19orf25	19	1469263	C	T	B3N: HTZ	SNV	downstream gene variant	unknown	46	50	rs1010420988	
		1469263	C	T	B3R: HTZ	SNV	downstream gene variant	unknown	47	63	rs1010420988	
		1483946	G	GGGGGGC	A1N: HTZ	INS	upstream gene variant	unknown	47	21	rs770134162	
		1483946	G	GGGGGGC	A1R: HTZ	INS	upstream gene variant	unknown	39	33	rs770134162	
		1483946	G	GGGGGGC	A5N: HTZ	INS	upstream gene variant	unknown	52	21	rs770134162	
		1483946	G	GGGGGGC	A5R: HTZ	INS	upstream gene variant	unknown	35	20	rs770134162	
		1484141	G	C	B3N: HTZ	SNV	upstream gene variant	unknown	63	68	rs375670143	
		1484141	G	C	B3R: HTZ	SNV	upstream gene variant	unknown	59	104	rs375670143	
CACNA1I	22	39663711	T	C	A5R: HTZ	SNV	splice region variant and intron variant	low	21	28	rs1359196258	5.6%
CALML4	15	68194078	G	GGGTTCGATATCTGCTTCC CTGAAGAGATCATCCACTG CAATAAATCACATTTAATTT TTCAGTTTGGCTTTAGT	B4R: HTZ	INS	stop gained and frameshift variant	high	20	20		
CAMK2D	4	113465607	A	T	B2R: HTZ	SNV	splice region variant and intron variant	low	26	26		
		113465609	A	T	B2R: HTZ	SNV	splice region variant and intron variant	low	26	26		
CAND1	12	67299119	T	TCATCATCGTAATTATAA	B2R: HTZ	INS	intron variant	unknown	36	11		
CCDC141	2	178961495	T	A	B2R: HTZ	SNV	intron variant	unknown	20	10	rs1274710229	5.6%
CD2AP	6	47503453	A	ACATTCCTGAT	B2R: HTZ	INS	intron variant	unknown	21	19		
CEP57	11	95813608	A	C	B2R: HTZ	SNV	intron variant	unknown	20	10	rs777827395	
		95813609	G	C	B2R: HTZ	SNV	intron variant	unknown	20	10		
		95813611	TTGATACTC	T	B2R: HTZ	DEL	intron variant	unknown	20	10		
CFAP54	12	96564786	C	CTTTA	A3R: HTZ	INS	intron variant	unknown	27	11		
		96564786	C	CTTTA	B2N: HTZ	INS	intron variant	unknown	36	11		
		96564786	C	CTTTA	B2R: HOMref	INS	intron variant	unknown	0	3		

CFAP61	20	20191418	T	A	B2R: HTZ	SNV	missense variant and splice region variant	moderate	25	16		6.9%
		20191420	G	A	B2R: HTZ	SNV	splice donor variant	high	23	17		
		20191423	A	AGCATAT	B2R: HTZ	INS	splice region variant and intron variant	low	23	17		
CFHR4	1	196918416	G	A	B2R: HTZ	SNV	3' UTR variant	unknown	20	10		
		196918418	T	A	B2R: HTZ	SNV	3' UTR variant	unknown	20	10		
		196918419	A	T	B2R: HTZ	SNV	3' UTR variant	unknown	20	10		
CPEB4	5	173944950	C	T	B2R: HTZ	SNV	intron variant	unknown	20	25	rs1357806020	
		173944951	C	T	B2R: HTZ	SNV	intron variant	unknown	20	25	rs765273202	
		173944953	G	T	B2R: HTZ	SNV	intron variant	unknown	20	24	rs1320811796	
		173944954	C	T	B2R: HTZ	SNV	intron variant	unknown	20	25	rs1366839043	
CRYBG1	6	106568576	G	GAAAATAATAA	B2R: HTZ	INS	3' UTR variant	unknown	33	12		
		106568581	A	T	B2R: HTZ	SNV	3' UTR variant	unknown	33	12		
CWF19L2	11	107441627	TA	T	B2R: HTZ	DEL	splice region variant and intron variant	low	35	14	rs749129275	
CYB5R4	6	83918059	A	ATAGT	B2R: HTZ	INS	frameshift variant	high	20	10		
		83918061	C	CATCTA	B2R: HTZ	INS	frameshift variant	high	20	10		
		83918065	G	A	B2R: HTZ	SNV	missense variant and splice region variant	moderate	20	10	rs758410641	
CYFIP1	15	22866869	A	AGCTT	B2R: HTZ	INS	downstream gene variant	unknown	20	10		
CYP26A1	10	93074148	G	C	B2R: HTZ	SNV	intron variant	unknown	25	12		
DCDC2	6	24302058	G	A	B2R: HTZ	SNV	intron variant	unknown	20	15	rs1240339666	
		24302059	G	A	B2R: HTZ	SNV	intron variant	unknown	20	15	rs200146922	
DDX4	5	55813763	G	GACAATGAAAT	B2R: HTZ	INS	stop gained and frameshift variant	high	23	13		
DDX50	10	68911269	G	GAGAATGT	B2R: HTZ	INS	intron variant	unknown	23	13		
DDX52	17	37626880	T	A	B2R: HTZ	SNV	intron variant	unknown	25	12	rs368820081	
		37626882	G	A	B2R: HTZ	SNV	intron variant	unknown	25	12	rs759618108	
		37626885	ATTGC	A	B2R: HTZ	DEL	intron variant	unknown	25	12	rs1462164594	
DDX60	4	168272162	A	AAAAGATCAGATAT	B2R: HTZ	INS	intron variant	unknown	21	19		
DGKD	2	233354520	TGGC	T	A5R: HTZ	DEL	start lost and inframe deletion	high	20	10	rs1405456455	
		233354520			A4N, A4R, B2R: position not sequenced							
DIAPH3	13	59810932	ATTTTG	A	B2R: HTZ	DEL	intron variant	unknown	20	15		
DIDO1	20	62896400	G	A	A3R: HTZ	SNV	splice region variant and intron variant	low	27	18		7.5%

DLD	7	107903548	G	GATCAAATTATCAAA	B2R: HTZ	INS	splice donor variant	high	20	10		
DMXL1	5	119129447	A	ATGCAGTTC	B2R: HTZ	INS	intron variant	unknown	38	13		5.2%
DNA2	10	68419203	C	CAAAAGTATTG	B2R: HTZ	INS	frameshift variant	high	21	14		
DNAH1	3	52392708	A	C	B4R: HTZ	SNV	intron variant	unknown	28	14	rs765661545	
		52392712	A	C	B4R: HTZ	SNV	intron variant	unknown	30	13	rs753193553	
DNAJC18	5	139422820	G	GTTCT	A3R: HTZ	INS	splice region variant and intron variant	low	25	12		
		139422822	A	T	A3R: HTZ	SNV	splice region variant and intron variant	low	25	12		
		139422825	A	ATC	A3R: HTZ	INS	intron variant	unknown	27	11		
DOPEY1	6	83130422	G	A	B2R: HTZ	SNV	intron variant	unknown	30	10		
DYNLRB1	20	34540563	C	T	B2R: HTZ	SNV	downstream gene variant	unknown	25	12	rs1264981462	
		34540564	A	T	B2R: HTZ	SNV	intron variant	unknown	25	12	rs1475627348	
EFCAB5	17	30034220	TCCCC	T	B2R: HTZ	DEL	splice region variant and intron variant	low	21	14		
		30034224	C	CTTTT	B2R: HTZ	INS	splice region variant and intron variant	low	21	14	rs1353716156	
		30034226	G	T	B2R: HTZ	SNV	splice region variant and intron variant	low	21	14	rs1290150982	
		30034228	A	T	B2R: HTZ	SNV	splice acceptor variant	high	21	14	rs1215962897	
EPB41	1	29058641	C	G	B4N: HTZ	SNV	missense variant	moderate	50	155	rs149962963	
		29058641	C	G	B4R: HTZ	SNV	missense variant	moderate	49	128	rs149962963	
		29109462	T	G	A3N: HTZ	SNV	intron variant	unknown	45	42	rs191910217	
		29109462	T	G	A3R: HTZ	SNV	intron variant	unknown	60	50	rs191910217	
		29109462	T	G	A5N: HTZ	SNV	intron variant	unknown	35	53	rs191910217	
		29109462	T	G	A5R: HTZ	SNV	intron variant	unknown	58	58	rs191910217	
ERCC5	13	102868282	T	TTTATCAA	B2R: HTZ	INS	intron variant	unknown	30	13	rs772409511	
ERP27	12	14915705	G	GAAAAA	B2R: HTZ	INS	intron variant	unknown	21	14		
		14915709	G	A	B2R: HTZ	SNV	intron variant	unknown	25	12	rs897087781	
FAM208B	10	5746167	TGGCCG	T	A3R: HTZ	DEL	splice region variant and intron variant	low	23	13		
FBXL21	5	135940677	A	G	B3R: HTZ	SNV	non transcrit coding exon variant	unknown	22	18		
FCER2	19	7689169	C	A	B2N: HTZ	SNV	3' UTR variant	unknown	34	41	rs142426433	
		7689169	C	A	B2R: HTZ	SNV	3' UTR variant	unknown	51	41	rs142426433	
		7689169	C	A	B3N: HTZ	SNV	3' UTR variant	unknown	54	57	rs142426433	
		7689169	C	A	B3R: HTZ	SNV	3' UTR variant	unknown	64	88	rs142426433	
FGFR1OP	6	167022388	T	C	B2R: HTZ	SNV	intron variant	unknown	20	25		

FRS2	12	69571429	C	CACATTTA	B2R: HTZ	INS	frameshift variant	high	20	25		
FRZB	2	182838020	TTGAAAGAAGCAAAC	T	B2R: HTZ	DEL	intron variant	unknown	31	16		
GABRA2	4	46337106	A	ATTTT	B2R: HTZ	INS	intron variant	unknown	20	15		7.2%
GFM2	5	74726142	A	AACATCATCTG	B2R: HTZ	INS	intron variant	unknown	21	14		
GLRA3	4	174659220	G	GAA	B2R: HTZ	INS	intron variant	unknown	23	13	rs1177909268	
GNRHR	4	67740732	A	ACTCCCTACTATG	B2R: HTZ	INS	intron variant	unknown	20	20		
GOLGA6L2	15	23439917	A	ACATCTTCTCCTCCTGGCCCCGCATCTTCTCCTCCTGCTCCCG	A4R: HTZ	INS	inframe insertion	moderate	48	54	rs773823433	
GPR141	7	37741317	A	G	B2R: HTZ	SNV	3' UTR variant	unknown	30	13		
GPSM2	1	108904207	A	ACATTTATATTTTAGAGTAG	B2R: HTZ	INS	stop gained and frameshift variant	high	23	34		
GRAMD1C	3	113844665	ACG	A	B2R: HTZ	DEL	intron variant	unknown	22	27		
		113844668	T	A	B2R: HTZ	SNV	intron variant	unknown	24	25		
HAVCR1	5	157037377	C	A	B2R: HTZ	SNV	intron variant	unknown	21	14	rs374244803	
		157037379	G	A	B2R: HTZ	SNV	intron variant	unknown	21	14	rs1313828880	
HEXB	5	74726142	A	AACATCATCTG	B2R: HTZ	INS	downstream gene variant	unknown	21	14		
IBTK	6	82225666	T	TGAAGATGGATG	B2R: HTZ	INS	intron variant	unknown	20	15		
ICE2	15	60476184	T	TATGTTTATTTGG	B2R: HTZ	INS	intron variant	unknown	23	17		
		60476188	C	T	B2R: HTZ	SNV	intron variant	unknown	25	16	rs552284423	
IFT81	12	110163064	A	ATAGATGCCT	B2R: HTZ	INS	protein altering variant and splice region variant	moderate	20	24		
IL6ST	5	55969867	C	A	B2R: HTZ	SNV	intron variant	unknown	20	10		
		55969868	A	AATGTATGGATT	B2R: HTZ	INS	intron variant	unknown	20	10		
IMPG2	3	101246113	G	GAAAAAAAAAA	B2R: HTZ	INS	intron variant	unknown	20	24		5.1%
IP6K2	3	48691463	ATTC	A	B2R: HTZ	DEL	inframe deletion	moderate	20	10	rs753889349	
IQGAP1	15	90494851	T	TCTTTCCTTTCA	B2R: HTZ	INS	intron variant	unknown	29	17		
KCTD3	1	215573762	ATACC	A	B2R: HTZ	DEL	intron variant	unknown	21	19		
KIF15	3	44775248	C	T	A3R: HTZ	SNV	splice region variant and intron variant	low	21	42		
KNTC1	12	122621960	T	TAATAGAAAATTGTTATTATTTA	B2R: HTZ	INS	frameshift variant	high	23	43		
LCMT1	16	25164586	C	T	B2R: HTZ	SNV	intron variant	unknown	20	40	rs1468144803	
		25164589	A	T	B2R: HTZ	SNV	intron variant	unknown	20	40	rs1482603426	

LNPEP	5	97003555	C	CAA	B2R: HTZ	INS	intron variant	unknown	20	10	
MAATS1	3	119732499	A	ATAATCCTTGATGAT	A3R: HTZ	INS	intron variant	unknown	22	18	
MAP3K2	2	127326832	A	AG	B2R: HTZ	INS	intron variant	unknown	25	12	
		127326833	T	TTTGATTTTTTA	B2R: HTZ	INS	intron variant	unknown	25	12	
MCM8	20	5968040	A	C	B2R: HTZ	SNV	intron variant	unknown	20	20	
MECR	1	29202019	C	T	A3N: HTZ	SNV	missense variant	moderate	60	66	rs11544658
		29202019	C	T	A3R: HTZ	SNV	missense variant	moderate	49	89	rs11544658
		29202019	C	T	A4N: HTZ	SNV	missense variant	moderate	49	83	rs11544658
		29202019	C	T	A4R: HTZ	SNV	missense variant	moderate	48	88	rs11544658
		29202019	C	T	A5N: HTZ	SNV	missense variant	moderate	37	99	rs11544658
		29202019	C	T	A5R: HTZ	SNV	missense variant	moderate	46	90	rs11544658
MED1	17	39423814	A	ATCTAGTACTTGTT	B2R: HTZ	INS	stop gained and frameshift variant	high	20	20	
MMP7	11	102523393	A	ACTATATG	B2R: HTZ	INS	frameshift variant	high	20	50	
		102523395	T	TAA	B2R: HTZ	INS	frameshift variant	high	20	50	
MTFR2	6	136239478	C	T	B2N: HTZ	SNV	missense variant	moderate	52	17	rs145047825
		136239478	C	T	B2R: HTZ	SNV	missense variant	moderate	47	23	rs145047825
		136239478	C	T	B3N: HTZ	SNV	missense variant	moderate	41	74	rs145047825
		136239478	C	T	B3R: HTZ	SNV	missense variant	moderate	34	32	rs145047825
NAA25	12	112040577	A	ATTGTTGT	B2R: HTZ	INS	frameshift variant and splice region variant	high	20	15	
		112040579	C	CTTAA	B2R: HTZ	INS	splice acceptor variant	high	20	15	
NAMPT	7	106251207	A	AGCAGCAC	B2R: HTZ	INS	intron variant	unknown	30	10	
NCKAP1	2	182952527	A	AGCTTTTAAACAT	B2R: HTZ	INS	intron variant	unknown	30	10	rs1424818952
NDUFB10	16	1961643	G	GGCCC	A4R: HTZ	INS	splice region variant and intron variant	low	21	33	
NIPA2	15	22866869	A	AGCTT	B2R: HTZ	INS	3' UTR variant	unknown	20	10	
NKX2-3	10	99535434	TCGGCCGCGGCCGCCGCCGCCGCCGCCGCCGCCGCCGCAGCAGCGGCGGCCTACAGCAGCAGCTATGGCTGTGCGTACCCGGCGGGCGGCGG	T	A4R: HTZ	DEL	frameshift variant	high	21	76	
NNMT	11	114296719	G	GA	B2R: HTZ	INS	intron variant	unknown	20	10	
		114296720	T	TTCCATTA	B2R: HTZ	INS	intron variant	unknown	20	10	

NLRP8	19	55954767	C	T	B2N: HTZ	SNV	missense variant	moderate	38	119	rs148518241	
		55954767	C	T	B2R: HTZ	SNV	missense variant	moderate	48	100	rs148518241	
		55954767	C	T	B3N: HTZ	SNV	missense variant	moderate	48	131	rs148518241	6.8%
		55954767	C	T	B3R: HTZ	SNV	missense variant	moderate	46	186	rs148518241	
NOBOX	7	144399085	G	T	A3R: HTZ	SNV	missense variant	moderate	30	10		
NOSTRIN	2	168834205	G	T	B2R: HTZ	SNV	intron variant	unknown	20	10	rs770185386	
		168834209	G	T	B2R: HTZ	SNV	intron variant	unknown	20	10	rs1171873320	
		168834211	A	T	B2R: HTZ	SNV	intron variant	unknown	20	10	rs1394605777	
NSMAF	8	58599320	G	GTAATATT	B2R: HTZ	INS	frameshift variant	high	20	25		
NUP98	11	3725195	C	CTTAGTAGAGAGTT	B2R: HTZ	INS	stop gained and frameshift variant	high	25	12		
NUP107	12	68732627	TAA	T	B2R: HTZ	DEL	splice region variant and intron variant	low	40	15	rs1234378427	
		68732629	A	ATTTTTT	B2R: HTZ	INS	splice region variant and intron variant	low	40	15		
OR10AG1	11	55968485	T	TTAG	B2R: HTZ	INS	upstream gene variant	unknown	33	12		
OVCH1	12	29444267	A	AATGGAAGAAGT	B2R: HTZ	INS	frameshift variant	high	20	20		7.2%
		29444268	C	G	B2R: HTZ	SNV	missense variant	moderate	21	19		
OXR1	8	106679309	C	T	B2R: HTZ	SNV	intron variant	unknown	33	15	rs374164784	
PCSK4	19	1483946	G	GGGGGGC	A1N: HTZ	INS	splice region variant and intron variant	low	47	21	rs770134162	
		1483946	G	GGGGGGC	A1R: HTZ	INS	splice region variant and intron variant	low	39	33	rs770134162	
		1483946	G	GGGGGGC	A5N: HTZ	INS	splice region variant and intron variant	low	52	21	rs770134162	
		1483946	G	GGGGGGC	A5R: HTZ	INS	splice region variant and intron variant	low	35	20	rs770134162	
		1484141	G	C	B3N: HTZ	SNV	intron variant	unknown	63	68	rs375670143	
		1484141	G	C	B3R: HTZ	SNV	intron variant	unknown	59	104	rs375670143	
PDE10A	6	165543578	A	ACTAAGCATTTAATAT	B2R: HTZ	INS	intron variant	unknown	20	15		6%
PIAS1	15	68194078	G	GGGTTCGATATCTGCTTCC<br>CTGAAGAGATCATCCACTG<br>CAATAAATCACATTTAATTT<br>TTCAGTTTGGCTTTAGT	B4R: HTZ	INS	downstream gene variant	unknown	20	20		
PLCL1	2	198083742	TCAAA	T	A3R: HTZ	DEL	intron variant	unknown	20	53	rs1350900552	6.4%
POC5	5	75707876	C	A	B2R: HTZ	SNV	splice acceptor variant	high	26	19		
		75707877	T	TAGTCAAGGTTATATATAT<br>TTTTC	B2R: HTZ	INS	splice region variant and intron variant	low	27	18		
POLH	6	43604601	G	T	B2R: HTZ	SNV	intron variant	unknown	23	13	rs769128596	

		43604602	C	T	B2R: HTZ	SNV	intron variant	unknown	23	13	rs1402343554	
		43604604	G	T	B2R: HTZ	SNV	intron variant	unknown	23	13	rs1415659711	
		43604605	G	T	B2R: HTZ	SNV	intron variant	unknown	21	13	rs1296324074	
PRUNE2	9	76854096	TCCCTCACC	T	B2R: HTZ	DEL	splice donor variant and coding sequence variant and intron variant	high	36	25	rs1359098284	10.9%
PSMB1	6	170546202	C	CTACACTATTTTAAATTAAT	B2R: HTZ	INS	intron variant	unknown	22	18		
PSPH	7	56015184	A	AAGTGAT	B2R: HTZ	INS	intron variant	unknown	20	20		
		56015189	A	C	B2R: HTZ	SNV	intron variant	unknown	21	19		
PTGES3	12	56666286	T	TCTTTTTTC	B2R: HTZ	INS	intron variant	unknown	22	18	rs1270418973	
PTPN13	4	86766529	T	TAAATAAAAATCCTACATG	B2R: HTZ	INS	intron variant	unknown	21	14		
RASA1	5	87363341	TAAACAG	T	A3R: HTZ	DEL	splice region variant and intron variant	high	23	17		
		87363347	G	GTTTTTT	A3R: HTZ	INS	protein altering variant and splice region variant	moderate	23	17		
RHOBTB3	5	95737097	T	TATCAC	B2R: HTZ	INS	intron variant	unknown	36	11		
RNGTT	6	88941175	A	AACAAAATTTAT	B2R: HTZ	INS	frameshift variant	high	25	20		
		88941177	C	T	B2R: HTZ	SNV	missense variant	moderate	22	18		
ROCK1	18	20959930	T	A	B2R: HTZ	SNV	splice acceptor variant	high	20	15	rs1357098673	
		20959931	G	A	B2R: HTZ	SNV	splice region variant and intron variant	low	20	15	rs1248294040	
		20959935	T	A	B2R: HTZ	SNV	splice region variant and intron variant	low	20	15	rs1273040681	
		20959938	G	A	B2R: HTZ	SNV	intron variant	unknown	21	14	rs1322185641	
RPA2	1	27897131	T	A	B2R: HTZ	SNV	intron variant	unknown	25	12	rs1225849655	
		27897132	G	A	B2R: HTZ	SNV	intron variant	unknown	27	11	rs1310056808	
		27897135	G	A	B2R: HTZ	SNV	intron variant	unknown	25	12	rs1333787495	
		27897136	G	A	B2R: HTZ	SNV	intron variant	unknown	25	12	rs1454019304	
RPS2	16	1961643	G	GGCCC	A4R: HTZ	INS	downstream gene variant	unknown	21	33		
RTN4IP1	6	106568576	G	GAAAATAATAA	B2R: HTZ	INS	downstream gene variant	unknown	33	12		
		106568581	A	T	B2R: HTZ	SNV	downstream gene variant	unknown	33	12		
SCFD1	14	30628292	C	CAAATATTGTCATTG	B2R: HTZ	INS	intron variant	unknown	20	15		
SEC23B	20	18515755	A	ATAACTATATTACACTT	B2R: HTZ	INS	intron variant	unknown	45	11		
SH2B2	7	102314385	G	C	A1N: HTZ	SNV	missense variant	moderate	50	114	rs1801728	
		102314385	G	C	A1R: HTZ	SNV	missense variant	moderate	48	158	rs1801728	
		102314385	G	C	A3N: HTZ	SNV	missense variant	moderate	40	69	rs1801728	

		102314385	G	C	A3R: HTZ	SNV	missense variant	moderate	50	88	rs1801728	
SLK	10	104020484	A	T	A3R: HTZ	SNV	splice region variant and intron variant	low	31	19	rs1317844509	
SRP72	4	56483280	T	TTCACTTTCTTCTTGG	B2R: HTZ	INS	intron variant	unknown	27	11		
TEKT5	16	10689237	A	C	B2R: HTZ	SNV	intron variant	unknown	27	11		
TMCO1	1	165743310	A	ATATGTATAT	B2R: HTZ	INS	inframe insertion and splice region variant	moderate	36	11		
TMEM209	7	130207928	C	T	B2R: HTZ	SNV	upstream gene variant	unknown	22	22	rs201225197	
TPH1	11	18033380	A	AGT	B2R: HTZ	INS	splice region variant and intron variant	low	21	14		
		18033381	A	ATTTTCTT	B2R: HTZ	INS	splice region variant and intron variant	low	21	14		
TRPC3	4	121903074	A	ATCATTTGTTT	B2R: HTZ	INS	intron variant	unknown	25	12		
UACA	15	70676597	A	AAATAGATTTAAATATTT	B2R: HTZ	INS	splice region variant and intron variant	low	21	19		
UBR2	6	42650398	A	AG	B2R: HTZ	INS	intron variant	unknown	30	10		
		42650399	A	AAAGGACC	B2R: HTZ	INS	intron variant	unknown	27	11		
UGDH	4	39500246	G	GTGA	B2R: HTZ	INS	inframe insertion	moderate	20	15		
USP53	4	119291145	C	A	B2R: HTZ	SNV	intron variant	unknown	30	13		
		119291151	C	A	B2R: HTZ	SNV	intron variant	unknown	28	14		
XPO1	2	61488781	A	ATTTATTTAGATATGCTTAATGT	B2R: HTZ	INS	intron variant	unknown	22	22		
ZBBX	3	167365999	A	AAAGG	A3R: HTZ	INS	intron variant	unknown	20	20	rs752870881	7%
ZNF263	16	3284207	T	TGA	A1R: HTZ	INS	splice region variant and intron variant	low	22	23	rs774271883	

[1] RefSeq GRCh38.p12 (GFC_000001405.38)

[2] According to the Ensembl Variant Effect Predictor (release VEP95.1)

[3] Subjective classification of the severity of the variant consequence

[4] Mutated genes in lung cancer. At least one mutation affecting more than 5% of the cases (Adenomas and adenocarcinomas, n=524; squamous cell neoplasms, n=490)

HTZ: Heterozygous variant; HOMref: Homozygous to RefSeq

High: The variant is assumed to have high (disruptive) impact in the protein, probably causing protein truncation, loss of function or triggering nonsense mediated decay / Moderate: A non-disruptive variant that might change protein effectiveness / Low: Assumed to be mostly harmless or unlikely to change protein behavior / Unknown: Usually non-coding variants or variants affecting non-coding genes, where predictions are difficult or there is no evidence of impact

3. Supporting information Figures

Figure S1. Sequencing data of non-COPD non-remodelled samples: major results reported for A1N, A3N, A4N, and A5N.

Histograms and pie charts showing quantitative data of identified genetic variations per sample.

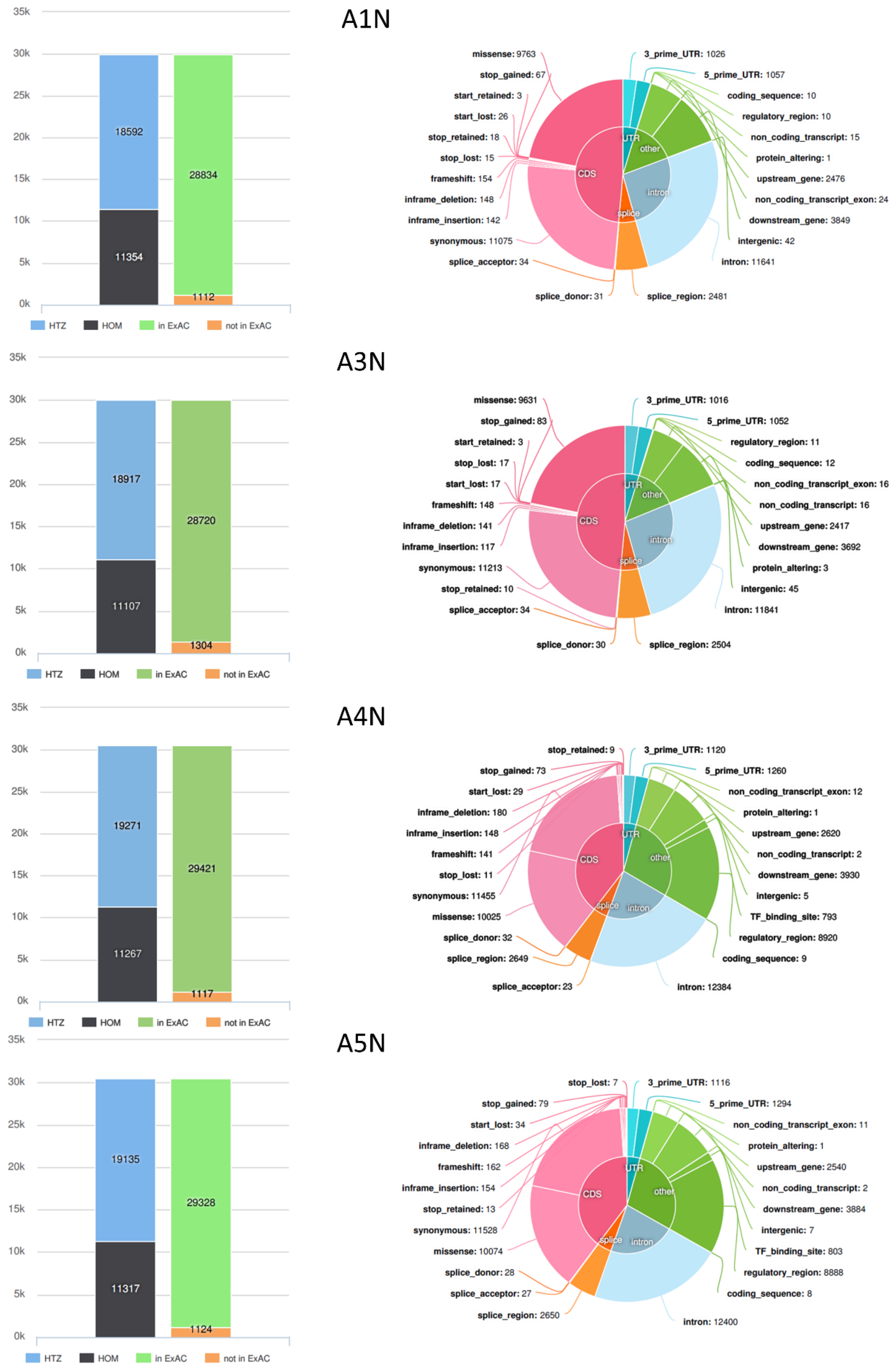

Figure S2. Sequencing data of non-COPD remodelled samples: major results reported for A1R, A3R, A4R, and A5R.

Histograms and pie charts showing quantitative data of identified genetic variations per sample.

A1R

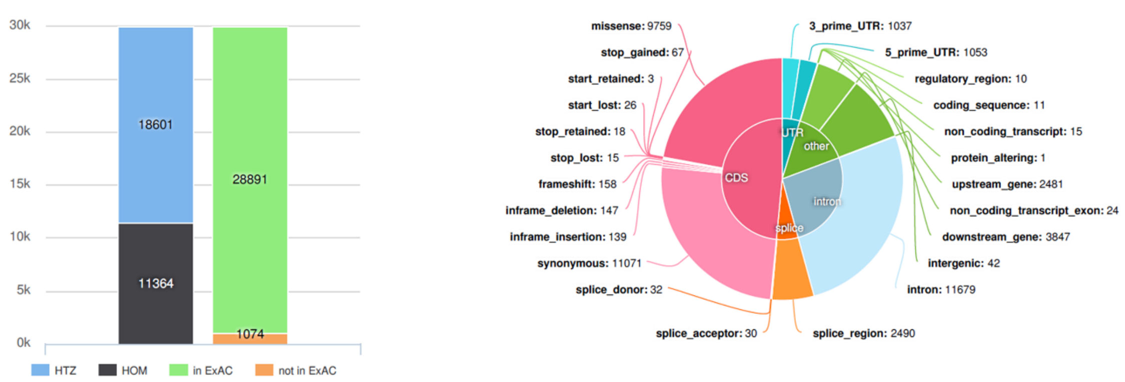

A3R

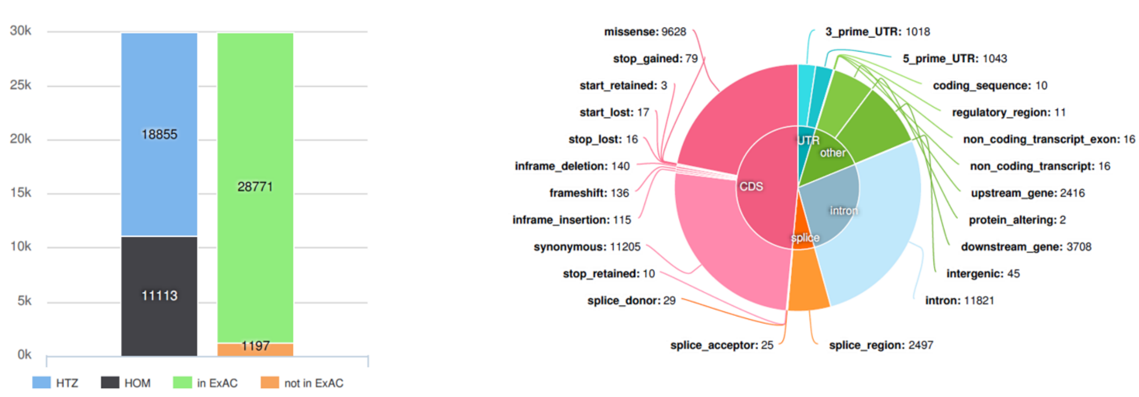

A4R

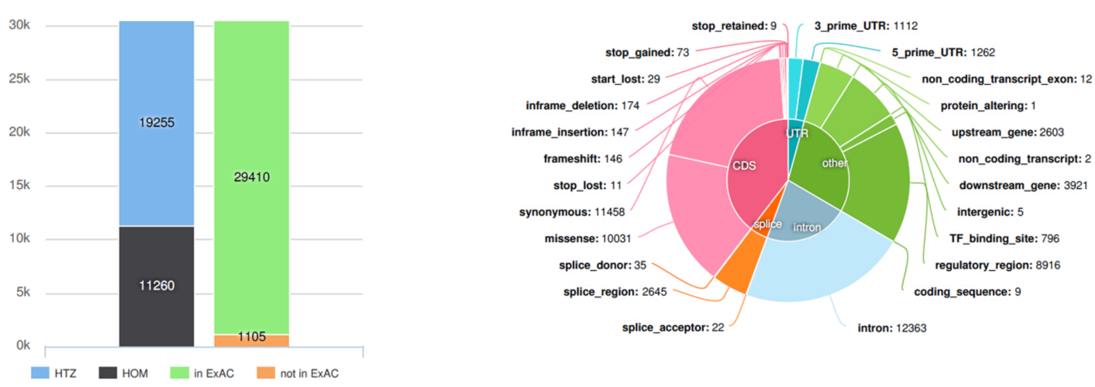

A5R

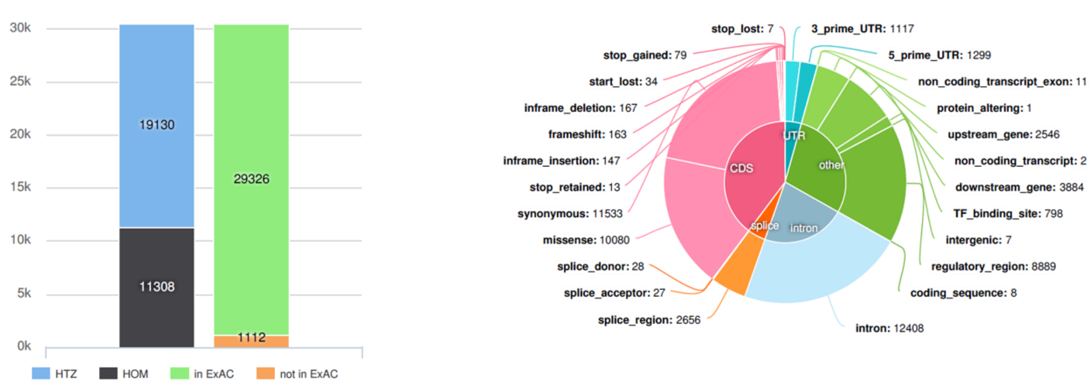

Figure S3. Sequencing data of COPD non-remodelled samples: major results reported for B2N, B3N, and B4N.

Histograms and pie charts showing quantitative data of identified genetic variations per sample.

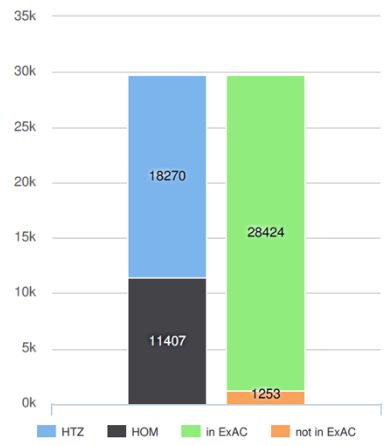

B2N

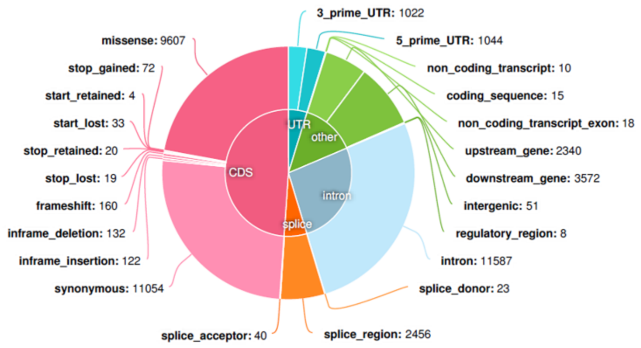

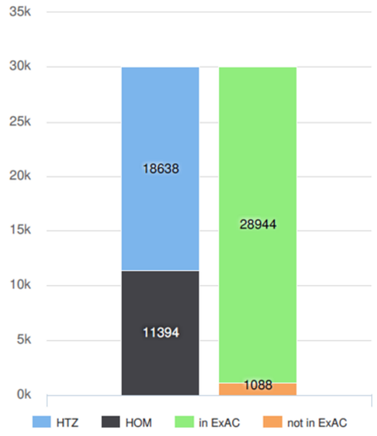

B3N

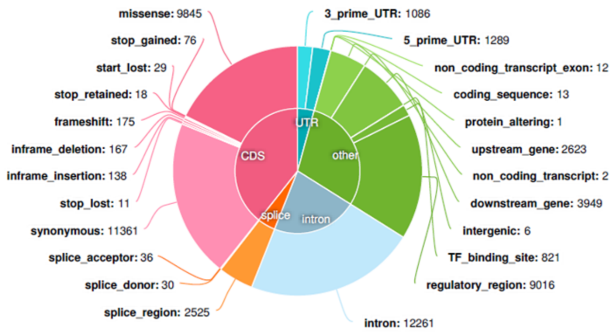

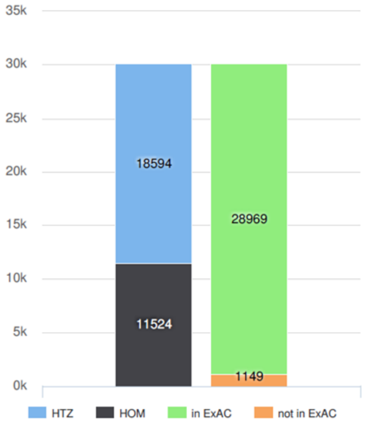

B4N

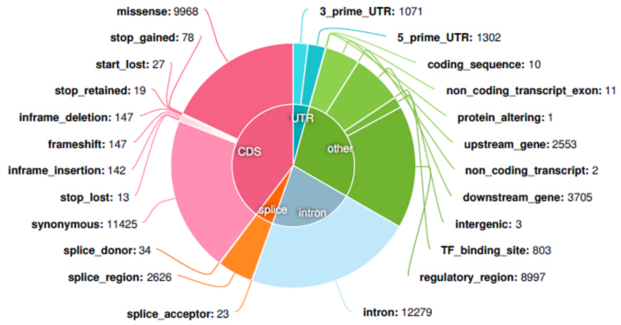

Figure S4. Sequencing data of COPD remodelled samples: major results reported for B2R, B3R, and B4R.

Histograms and pie charts showing quantitative data of identified genetic variations per sample.

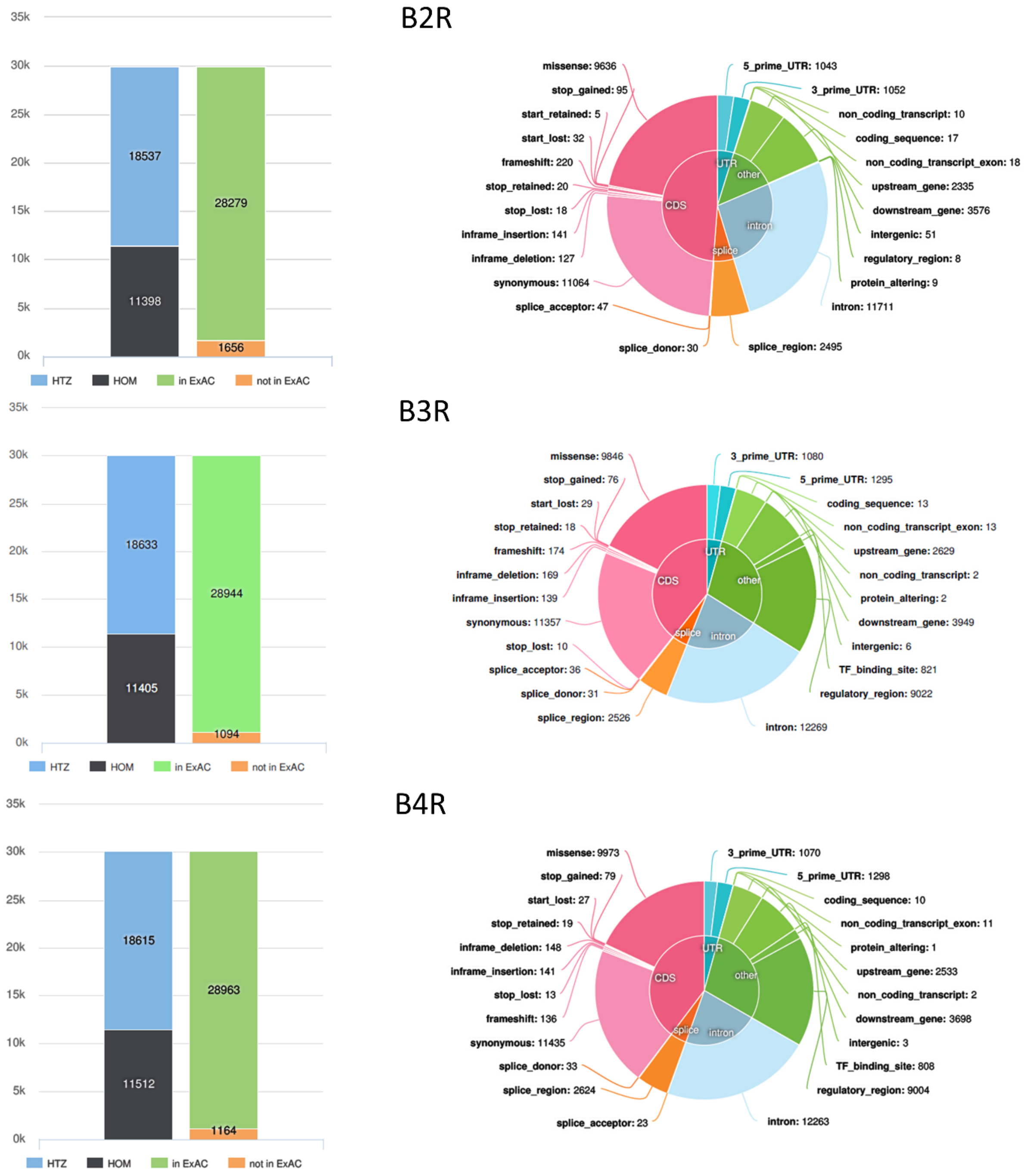

Figure S5. Protein network analysis related to the genes of interest having variants in remodelled COPD epithelia.

(A) Summary view showing known and predicted interactions (nodes and edges) for the input of 129/129 proteins. **(B)** Table reporting the categories and terms significantly associated with the network of proteins. Cilia-associated proteins are underlined.

A

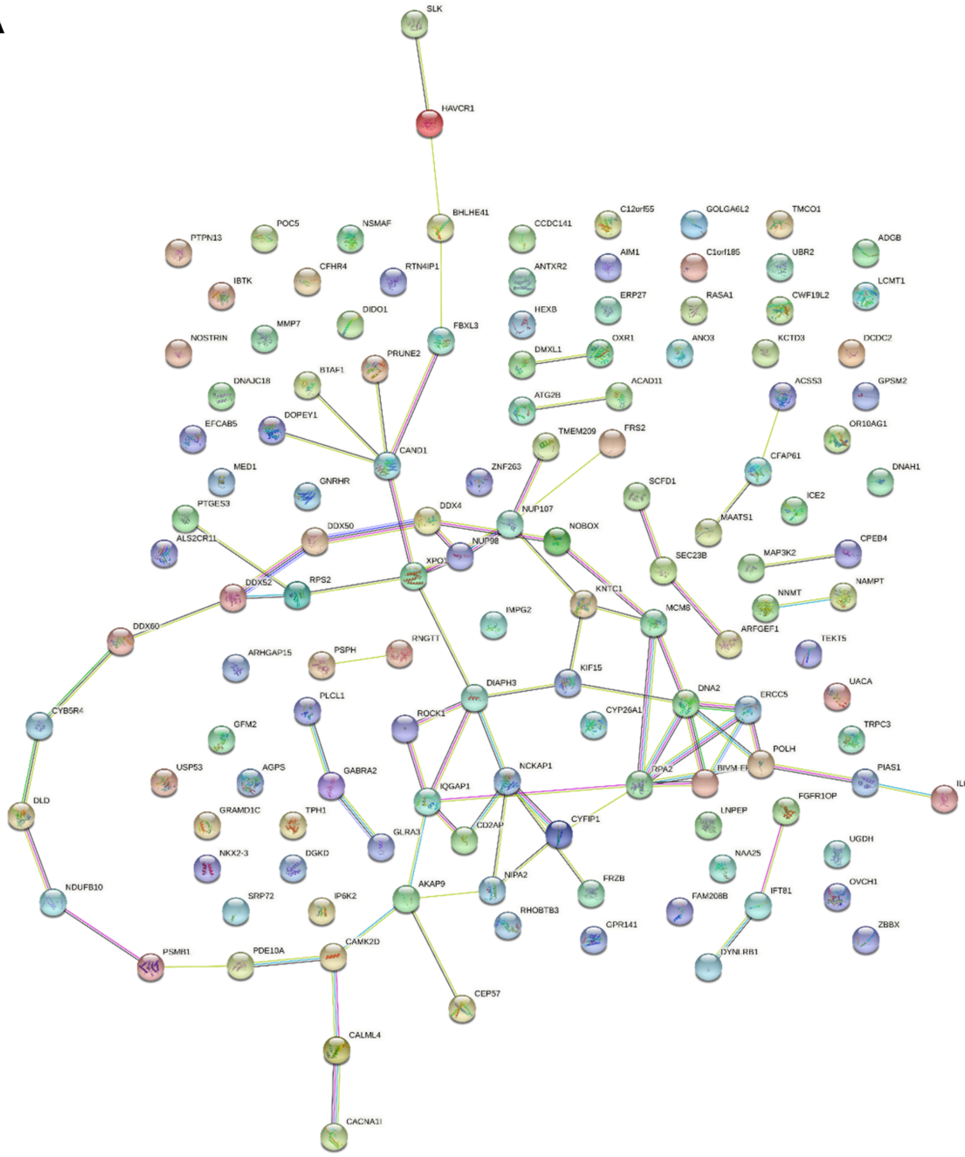

B

#category	term ID	term description	observed gene count	background gene count	strength	FDR	matching proteins in network
GO Process	GO:0003341	Cilium movement	8	130	0.97	0.0455	CFAP61,MAATS1,TEKT5,CACNA1I,ZBBX,DNAH1,DDX4,C12orf55
GO Process	GO:0007017	Microtubule-based process	17	727	0.55	0.0472	IFT81,CFAP61,MAATS1,TEKT5,CEP57,KIF15,AKAP9,DYNLRB1,FGFR1OP,SLK,CACNA1I,GPSM2,ZBBX,DNAH1,CALML4,DDX4,C12orf55
GO Process	GO:0030031	Cell projection assembly	13	450	0.64	0.0480	IFT81,TEKT5,CEP57,AKAP9,DYNLRB1,CD2AP,NCKAP1,FGFR1OP,DCDC2,ROCK1,DNAH1,C12orf55,CYFIP1
UniProt Keywords	KW-0347	Helicase	7	140	0.88	0.0354	BTAF1,DNA2,DDX50,MCM8,DDX60,DDX4,DDX52
UniProt Keywords	KW-0007	Acetylation	39	3341	0.25	0.0434	DLD,NAMPT,NUP107,LNPEP,IFT81,PIAS1,PSMB1,AGPS,ACAD11,DIDO1,IQGAP1,RASA1,MED1,UACA,NUP98,UGDH,KIF15,SEC23B,CAMK2D,RPS2,SRP72,ERCC5,DYNLRB1,DNA2,NCKAP1,CYB5R4,UBR2,RPA2,PRUNE2,PSPH,ROCK1,XPO1,SCFD1,POC5,NNMT,CAND1,ACSS3,DDX52,PTGES3
UniProt Keywords	KW-0966	Cell projection	18	1009	0.43	0.0434	IMPG2,DLD,IFT81,CFAP61,CPEB4,MAATS1,GLRA3,TEKT5,CD2AP,NCKAP1,FGFR1OP,DCDC2,ROCK1,PTPN13,DNAH1,GABRA2,C12orf55,CYFIP1

Figure S6. Heat map of CNA identified in microdissected epithelia.
The colours represent the number of DNA copies for the CNA.

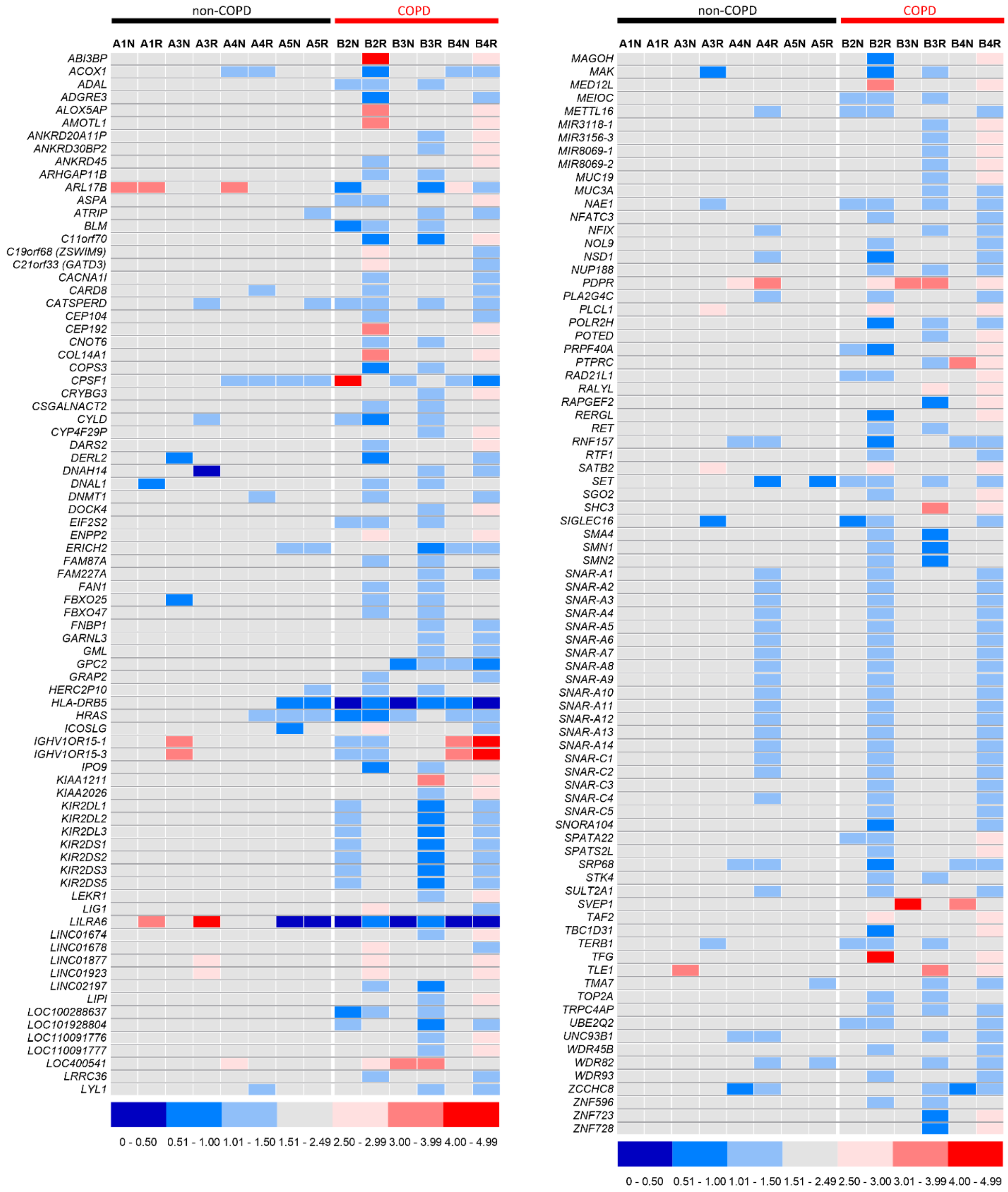

Figure S7. Protein network analysis related to the genes of interest having CNA in at least two samples in remodelled COPD epithelia.

(A) Summary view showing known and predicted interactions (nodes and edges) for the input of 110/160 proteins. **(B)** Table reporting the categories and terms significantly associated with the network of proteins.

A

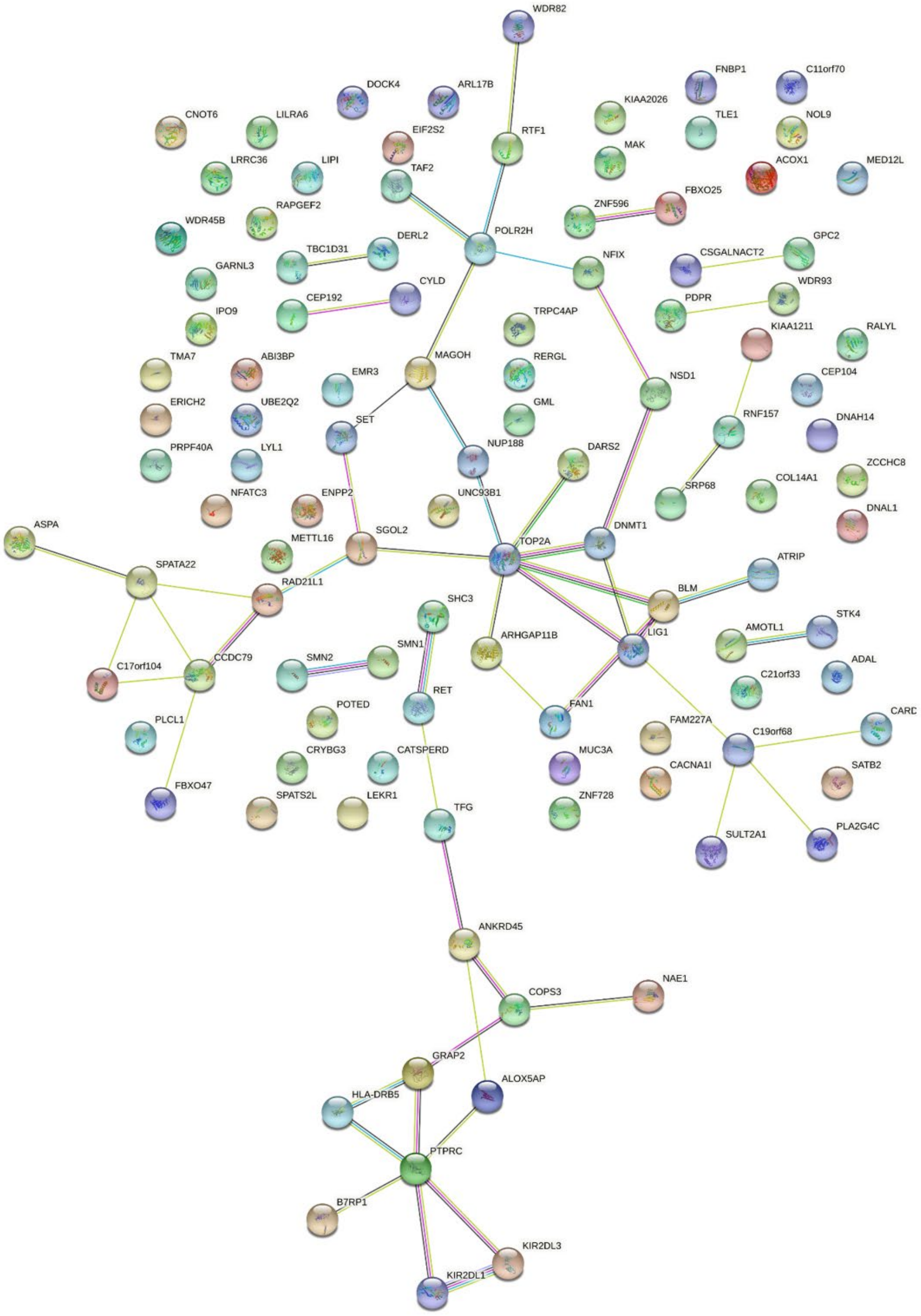

B

#category	term ID	term description	observed gene count	background gene count	strength	FDR	matching proteins in network
COMPARTMENTS	GOCC:0031981	Nuclear lumen	25	1791	0.39	0.0418	LIG1,PDPR,WDR82,NFATC3,SRP68,ATRIP,BLM,DNMT1,SET,STK4,TLE1,NOL9,TAF2,SMN1,SMN2,RTF1,RAD21L1,PRPF40A,NSD1,SATB2,TOP2A,POLR2H,MED12L,ZCCHC8,SPATS2L

Figure S8. Heat map of variants/CNA identified in microdissected epithelia.
For each gene, the first line corresponds to the CNA and the second line to the variants. The colours represent the number of DNA copies for the CNA. The grayscale is for the variant impact on the protein (crossed when the quality of sequencing is not satisfactory (GQ<20)).

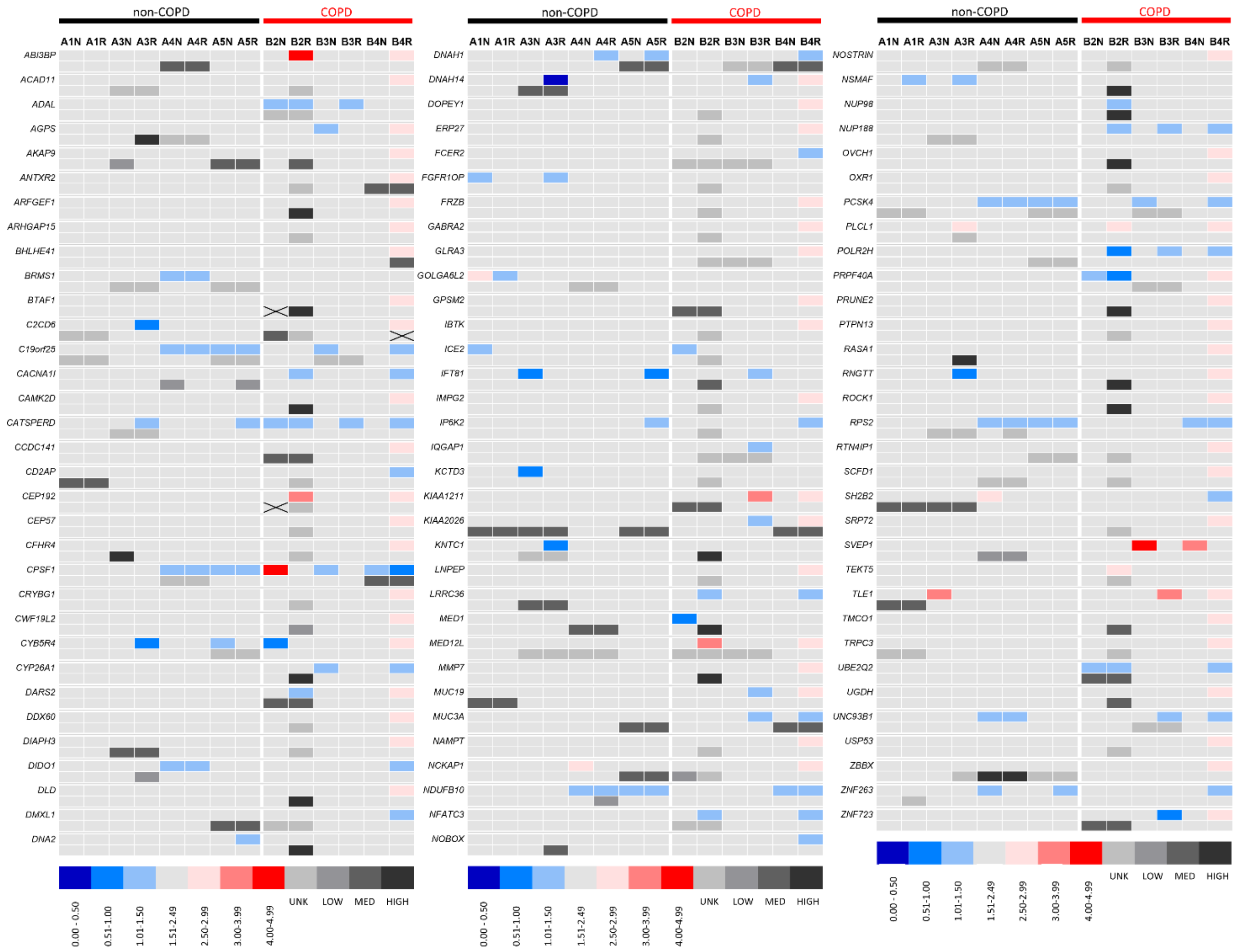

Figure S9. Protein network analysis related to the list of genes displaying variants and/or CNA in remodelled COPD epithelia.

(A) Summary view showing known and predicted interactions (nodes and edges) for the input of 237/269 proteins. **(B)** Table reporting the categories and terms significantly associated with the network of proteins. Cilia-associated proteins are underlined.

A

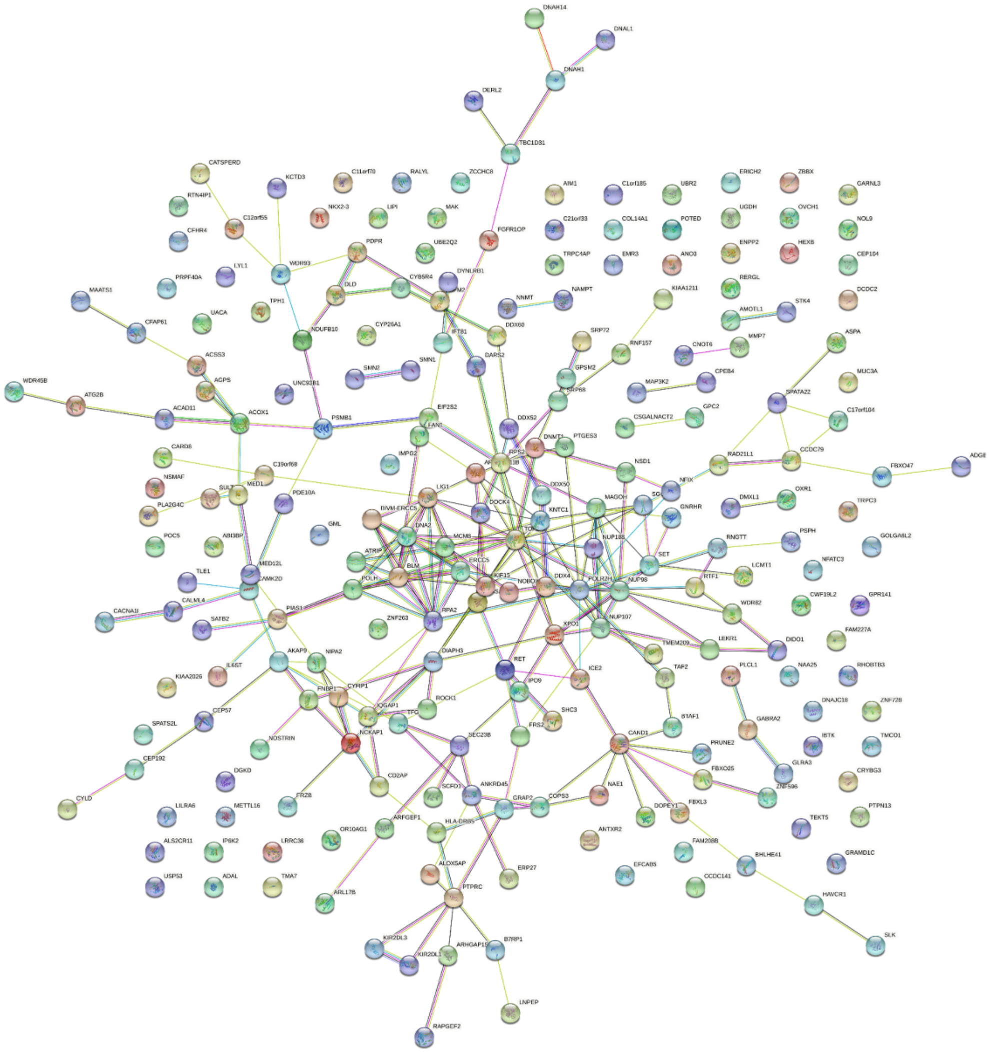

B

#category	term ID	term description	observed gene count	background gene count	strength	FDR	matching proteins in network
Reactome	HSA-1640170	Cell Cycle	22	647	0.45	0.0416	NUP107,PSMB1,LIG1,DIDO1,NUP98,CEP57,ATRIP,KNTC1,BLM,AKAP9,SGOL2,DNA2,FGFR1OP,NUP188,SET,RPA2,MCM8,LCMT1,XPO1,TOP2A,POLR2H,CEP192
TISSUES	BTO:0000081	Reproductive system	105	6107	0.15	0.0339	DLD,UNC93B1,NUP107,LNPEP,TFG,CFAP61,TRPC4AP,KCTD3,ENPP2,MMP7,HEXB,ICE2,NAA25,PSMB1,ARFGEF1,METTL16,LIG1,ACAD11,DIDO1,UBE2Q2,IQGAP1,NDUFB10,MAATS1,RASA1,TEKT5,PDPR,ACOX1,FRZB,COL14A1,POTED,MED1,ANTXR2,SRP68,NUP98,CEP57,UGDH,ATRIP,KIF15,IP6K2,NIPA2,SEC23B,RPS2,FBXL3,RET,DYNLRB1,SGOL2,GRAMD1C,NAE1,CD2AP,DNMT1,NCKAP1,IPO9,DARS2,FGFR1OP,AIM1,CYB5R4,SLK,MAGOH,NUP188,SET,UBR2,EIF2S2,TLE1,TAF2,MCM8,RHOBTB3,SMN1,SMN2,IL6ST,FBXO25,CRYBG3,RTF1,DDX60,EFCAB5,TMEM209,NFIX,LCMT1,DIAPH3,XPO1,C17orf104,PRPF40A,MAP3K2,AMOTL1,SCFD1,CYLD,NOSTRIN,OXR1,POC5,NSMAF,PTPRC,TOP2A,FNBP1,POLR2H,CALML4,KIAA1211,DDX4,ZCCHC8,CAND1,FRS2,SPATA22,DDX52,TMCO1,CYFIP1,PTGES3,SPATS2L
TISSUES	BTO:0000083	Female reproductive system	100	5799	0.15	0.0339	DLD,UNC93B1,NUP107,LNPEP,TFG,CFAP61,TRPC4AP,KCTD3,ENPP2,MMP7,HEXB,ICE2,NAA25,PSMB1,ARFGEF1,METTL16,LIG1,ACAD11,DIDO1,UBE2Q2,IQGAP1,NDUFB10,MAATS1,RASA1,TEKT5,PDPR,ACOX1,FRZB,COL14A1,MED1,ANTXR2,SRP68,NUP98,CEP57,UGDH,ATRIP,KIF15,IP6K2,NIPA2,SEC23B,RPS2,FBXL3,DYNLRB1,SGOL2,GRAMD1C,NAE1,CD2AP,DNMT1,NCKAP1,IPO9,DARS2,FGFR1OP,AIM1,CYB5R4,SLK,MAGOH,NUP188,SET,UBR2,EIF2S2,TLE1,TAF2,MCM8,RHOBTB3,SMN1,SMN2,IL6ST,FBXO25,CRYBG3,RTF1,DDX60,EFCAB5,TMEM209,LCMT1,DIAPH3,XPO1,C17orf104,PRPF40A,MAP3K2,AMOTL1,SCFD1,CYLD,NOSTRIN,OXR1,NSMAF,PTPRC,TOP2A,FNBP1,POLR2H,CALML4,KIAA1211,DDX4,ZCCHC8,CAND1,FRS2,SPATA22,TMCO1,CYFIP1,PTGES3,SPATS2L
TISSUES	BTO:0003091	Urogenital system	111	6716	0.13	0.0339	DLD,UNC93B1,NUP107,LNPEP,TFG,CFAP61,TRPC4AP,KCTD3,ENPP2,MMP7,HEXB,ICE2,NAA25,PSMB1,ARFGEF1,METTL16,LIG1,ACAD11,DIDO1,UBE2Q2,IQGAP1,NDUFB10,MAATS1,RASA1,TEKT5,PDPR,ACOX1,FRZB,COL14A1,POTED,MED1,NFATC3,ANTXR2,SRP68,NUP98,CEP57,UGDH,ATRIP,KIF15,IP6K2,NIPA2,SEC23B,RPS2,HAVCR1,FBXL3,RET,DYNLRB1,SGOL2,GRAMD1C,NAE1,CD2AP,DNMT1,NCKAP1,IPO9,DARS2,FGFR1OP,AIM1,CYB5R4,SLK,MAGOH,NUP188,SET,UBR2,RPA2,EIF2S2,TLE1,PRUNE2,TAF2,MCM8,RHOBTB3,SMN1,SMN2,IL6ST,FBXO25,CRYBG3,RTF1,CARD8,DDX60,EFCAB5,PSPH,TMEM209,NFIX,LCMT1,DIAPH3,XPO1,C17orf104,PRPF40A,MAP3K2,AMOTL1,SCFD1,CYLD,NOSTRIN,OXR1,POC5,NSMAF,PTPRC,TOP2A,FNBP1,POLR2H,CALML4,KIAA1211,DDX4,ZCCHC8,CAND1,FRS2,SPATA22,DDX52,TMCO1,CYFIP1,PTGES3,SPATS2L
UniProt Keywords	KW-0025	Alternative splicing	161	10179	0.12	0.00038	DLD,CYP26A1,GNRHR,NUP107,LNPEP,TFG,IFT81,CFAP61,PIAS1,TPH1,TRPC4AP,EMR3,ANO3,KCTD3,ENPP2,ICE2,NAA25,METTL16,LIG1,DGKD,ACAD11,CPEB4,BTAF1,DIDO1,UBE2Q2,WDR93,NDUFB10,COPS3,RNF157,MAATS1,GLRA3,RASA1,CWF19L2,ABI3BP,TBC1D31,PDPR,C21orf33,ACOX1,GFM2,COL14A1,MED1,NFATC3,IBTK,ANTXR2,SRP68,UACA,NUP98,CEP57,UGDH,ATRIP,KIF15,KNTC1,FAM208B,LRRC36,IP6K2,ANKRD45,KIR2DL1,NIPA2,GRAP2,B7RP1,CAMK2D,SRP72,KIR2DL3,LIPI,MAK,RET,ERCC5,FAM227A,AKAP9,DYNLRB1,SGOL2,GRAMD1C,DNA2,NAE1,DNMT1,NCKAP1,FAN1,FGFR1OP,CFHR4,RTN4IP1,AIM1,RNGTT,SLK,MAGOH,POLH,NUP188,SET,STK4,UBR2,GARNL3,RPA2,CSGALNACT2,SHC3,PRUNE2,CEP104,DCDC2,MCM8,MUC3A,TRPC3,SMN1,IL6ST,CATSPERD,FBXO25,CRYBG3,CARD8,EFCAB5,LILRA6,TMEM209,NFIX,ADGB,ZNF596,LCMT1,KIAA2026,DIAPH3,CACNA1I,RAD21L1,C17orf104,PRPF40A,AMOTL1,ZBBX,SCFD1,CYLD,PTPN13,NSD1,CCDC141,ADAL,SATB2,DNAH1,PLCL1,DOCK4,OXR1,ALS2CR11,POC5,NSMAF,PTPRC,TOP2A,FNBP1,C11orf70,POLR2H,MED12L,CALML4,NOBOX,GABRA2,DDX4,CEP192,RALYL,C12orf55,PDE10A,ZCCHC8,CAND1,ACSS3,DNAL1,GOLGA6L2,SPATA22,CCDC79,PLA2G4C,C19orf68,TMCO1,CYFIP1,PTGES3,SPATS2L
UniProt Keywords	KW-0007	Acetylation	67	3341	0.22	0.0049	DLD,NAMPT,NUP107,LNPEP,TFG,IFT81,PIAS1,TRPC4AP,PSMB1,LIG1,AGPS,ACAD11,DIDO1,IQGAP1,COPS3,RASA1,C21orf33,ACOX1,MED1,NFATC3,SRP68,UACA,NUP98,UGDH,KIF15,SEC23B,GRAP2,CAMK2D,RPS2,SRP72,BLM,ERCC5,DYNLRB1,DNA2,NAE1,DNMT1,NCKAP1,IPO9,DARS2,CYB5R4,MAGOH,NUP188,SET,STK4,UBR2,RPA2,EIF2S2,PRUNE2,NOL9,SMN1,PSPH,ROCK1,XPO1,PRPF40A,SCFD1,POC5,TOP2A,FNBP1,POLR2H,ZCCHC8,NNMT,CAND1,ACSS3,DNAL1,DDX52,PTGES3,SPATS2L

Figure S10. Exploration of gene hits associated with remodelling in lung and COPD public data. **(A)** Volcano plot showing remodelled COPD-associated deregulated genes quantification and significant fold change in COPD whole-lung tissues. Red dots, p-value<0.05 and FDR<0.05; grey dots, FDR>0.05. **(B)** Representative micrographs showing the bronchial epithelia on FFPE lung tissues from the Human Protein Atlas and cellular landscape along the airways **(C)** in human lung analysed by single-cell RNA sequencing to display specific cluster assignment of epithelial and non-epithelial cells (the identification of each specific gene-expressing cells by t-distributed Stochastic Neighbour Embedding (tSNE) is shown) for RET, NSD1, COL14A1, ZNF728, and AMOTL1.

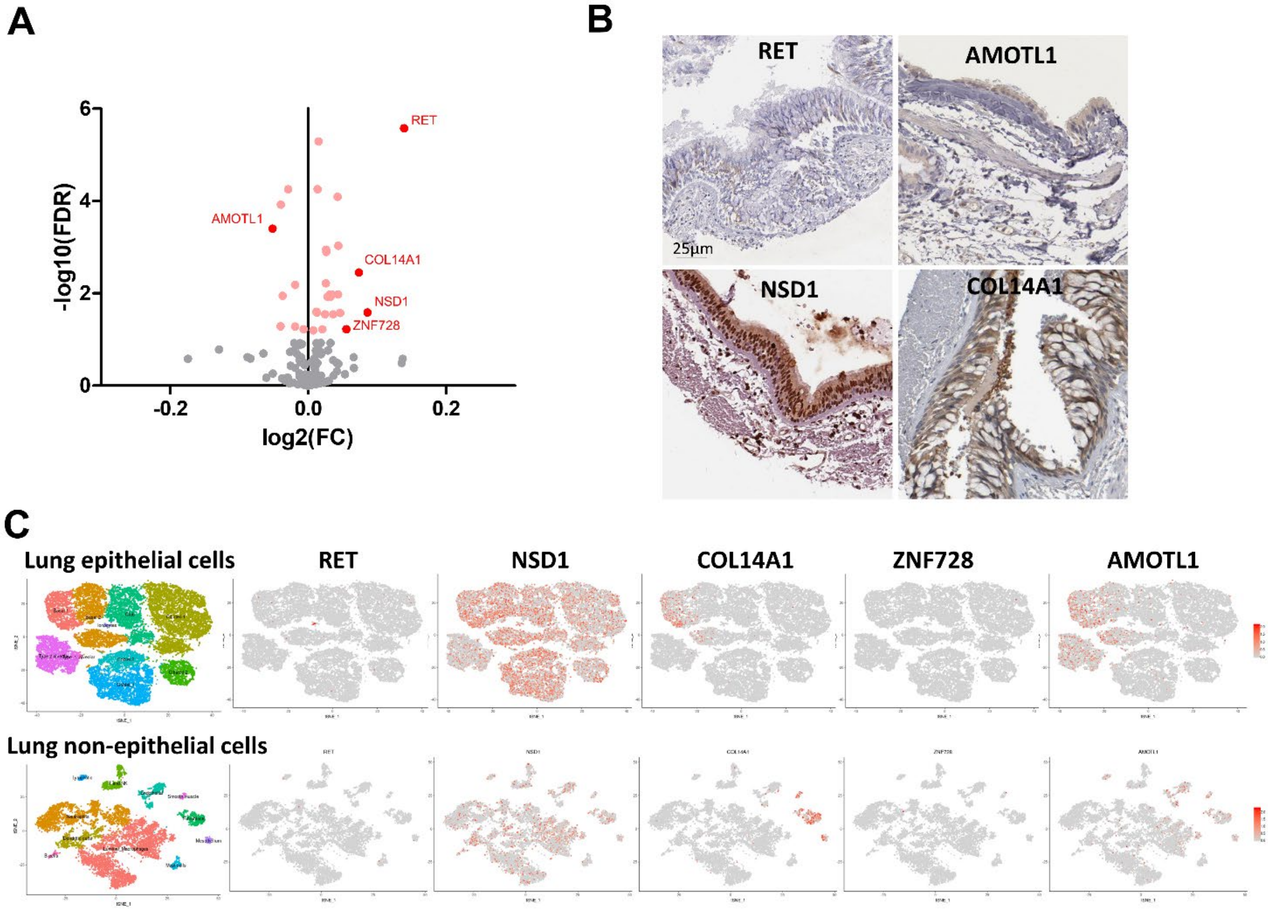

4. Additional References

1. Gnirke A, Melnikov A, Maguire J, Rogov P, LeProust EM, Brockman W, et al. Solution hybrid selection with ultra-long oligonucleotides for massively parallel targeted sequencing. Nat Biotechnol. 2009;27:182–9.
